# Hyperglycaemia-Induced Metabolic Stress Promotes EMT-Driven Therapeutic Resistance in Cancer: Evidence of a Deleterious Feed-Forward Cycle

**DOI:** 10.3390/ph19050769

**Published:** 2026-05-14

**Authors:** Rabia Zafar, Thanh Dat Pham, Lupeuea Vakafua, Teana Reed, Naisana Seyedasli

**Affiliations:** 1 School of Medical Sciences, Faculty of Medicine and Health, University of Sydney, Westmead Hospital, Westmead, NSW 2145, Australia; 2 Centre for Cancer Research, The Westmead Institute for Medical Research, Westmead, NSW 2145, Australia

**Keywords:** epithelial–mesenchymal transition (EMT), hyperglycaemia, metabolic stress, therapeutic resistance, cancer, carcinoma

## Abstract

The phenotypic plasticity of epithelial cells along the epithelial–mesenchymal (E-M) axis, or epithelial–mesenchymal transition (EMT), is a critical aspect of tumour progression and therapeutic resistance. During EMT, epithelial cells gradually acquire mesenchymal traits, facilitating vital functions in embryogenesis, wound healing, fibrosis, and tumour metastasis. This review article investigates the potential interplay between hyperglycaemia-induced metabolic stress and EMT in the context of therapeutic resistance. The study examines a complex, multifaceted network of molecular mechanisms regulating EMT, including specialised transcription factors and signalling pathways as well as growth factors, integrins, and matrix metalloproteinases in various epithelial carcinomas. Emerging findings have demonstrated the existence of EMT hybrid states along the continuum, possessing heightened metastatic potential and distinctive metabolic signatures that play critical roles in the development of therapeutic resistance in cancer cells. Hyperglycaemia has been particularly highlighted for its potential to promote EMT-driven therapeutic resistance through various interconnected mechanisms. Elevated glucose levels induce the increased production of reactive oxygen species (ROS), activation of EMT-promoting transcription factors, and a metabolic shift towards glycolysis. This hyperglycaemic stress involves upregulation of glucose transporters and glycolytic enzymes, creating feed-forward loops that support drug efflux mechanisms and help maintain the mesenchymal phenotype. Clinical data also indicate that hyperglycaemia in OSCC patients is associated with more advanced tumour stages, more extended hospital stays, less effective treatments, and higher rates of local recurrence and distant metastasis. Overall, these insights reveal a deleterious feed-forward loop in which hyperglycaemia promotes EMT-driven therapeutic resistance, with the strongest clinical evidence in oral squamous cell carcinoma (OSCC) and supportive data from pancreatic and breast cancers. Although glycaemic control represents a promising low-risk adjunctive approach, its clinical benefit remains to be validated in prospective interventional studies.

## 1. Introduction

**Figure 1:**
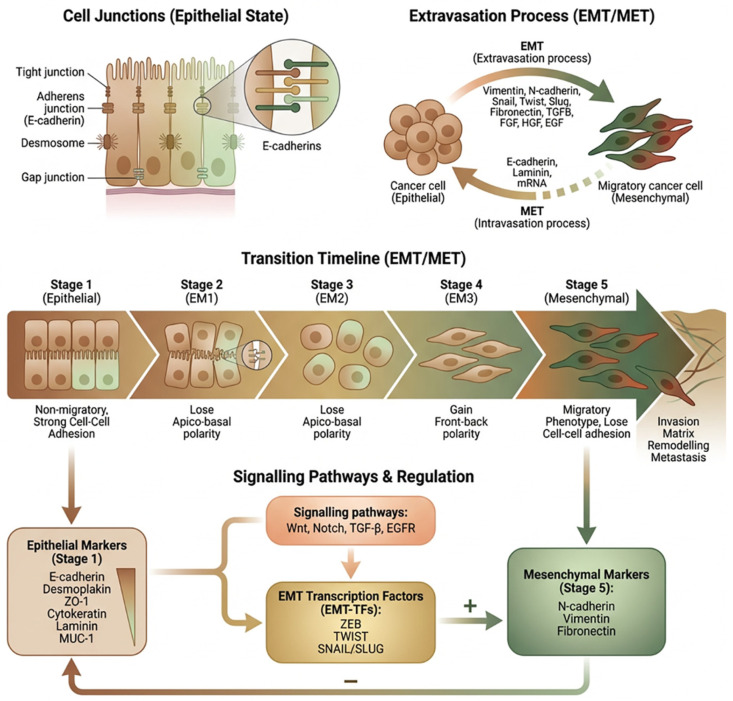
**Schematic overview of EMT and MET transitions.** The EMT process is stimulated by EMT-promoting transcription factors, leading to the loss of cell–cell junctions, apical–basal polarity, and epithelial properties and gain of mesenchymal phenotypes and behavioural changes that mediate invasive behaviour and facilitate migration. In some cases, cells can remain in a pseudo-EMT state, maintaining both epithelial and mesenchymal properties and behaviours. The reverse process is known as the mesenchymal-to-epithelial transition (MET). These transitions are further regulated by signalling pathways (created by GPAI).

The multifactorial transition of epithelial cells into mesenchymal cells is a process known as epithelial–mesenchymal transition (EMT), often characterised by the loss of cell adhesion and increased cell mobility [1,2]. It is a progressive and dynamic change in the cellular organisation of epithelial cells, in which they lose epithelial properties while acquiring the behaviours and phenotypes associated with mesenchymal cells [1,2]. Acquiring a mesenchymal phenotype enables cells to migrate to distant organs, maintain their stemness, and differentiate into multiple cell types, thereby initiating processes such as wound healing, metastasis, and fibrosis [1,3]. EMT is therefore associated with severe and aggressive stages of diseases, such as the generation of fibrosis and/or tumourigenesis [2,3,4]. The inductive cascade and transcriptional mediation of EMT are independent of changes in DNA and it is thus a reversible process [1,3]. In fact, the dynamic shifts between EMT and its reverse process, mesenchymal-to-epithelial transition (MET), are essential for the development of organs and tissues [2,4]. The activation of this conserved and reversible process of EMT is dependent on microenvironmental signals and inductive cues that interact with epigenetic regulators [2,4,5], whereby epigenetic regulators mediate the expression of proteins involved in cell polarity, cytoskeleton assembly and disassembly, and extracellular matrix degradation as well as cell-to-cell contact and adhesion [4,5]. EMT is known to be a fluid process in which cells can maintain a pseudo-state of EMT, where the epithelial cells adopt both epithelial and mesenchymal phenotypes and behaviours [4,5]. The EMT process is typically marked by the loss of epithelial markers, such as E-cadherin and cytokeratin, and an increase in mesenchymal proteins, including Vimentin and N-cadherin [5,6,7] (Figure 1).

Signalling pathways, such as Transforming Growth Factor (TGF)-β, Wingless-related integration site (Wnt), Fibroblast Growth Factor (FGF), and Notch, induce the activation of EMT-promoting transcription factors, including Snail, Twist, and ZEB, resulting in changes in epithelial cells [4,5,6,7] (Figure 1). Moreover, there is an emerging role for complex epigenetic regulatory programmes governing EMT states [4,5]. In this review, we aim to first build an overview of the EMT process and the inductive factors and regulatory pathways mediating this cellular event while re-constructing the mechanistic links between EMT and the key microenvironmental stress factor “hyperglycaemia” in the development of therapy resistance in carcinomas.

## 2. EMT Classification

### 2.1. Type I EMT: During Implantation, Embryogenesis, and Organ Development

Type I EMT plays critical roles in generating multilayered tissues and facilitating wound healing, as well as for gastrulation in metazoans and the neural crest delamination in vertebrates [1,2] (Table 1). However, uncontrolled and dysfunctional EMT results in pathological conditions such as carcinogenesis and fibrosis [2]. Researchers argue that the genetically abnormal cells undergoing cancer-related EMT show reduced and/or altered sensitivity to standard growth regulatory signals, differentiating these events from EMT during embryogenesis [2,4,8]. During embryonic development, the dynamic fluidity of sequential stages of EMT and MET is a crucial element in the development of three-dimensional structures, organs, and differentiation of specialised cells [4,8,9,10]. It is dependent on multiple cell-to-cell signalling cascades to induce changes in the epithelial cells, while activating the transcription factors that promote EMT [11,12,13,14,15,16,17]. For instance, Snail, Twist, and ZEB inhibit epithelial genes and promote mesenchymal genes [12]. In fact, Snail has been observed to directly repress E-cadherin, an epithelial transmembrane component of adherens junctions that forms intercellular adhesion in epithelial cells [10].

### 2.2. Type II EMT: Associated with Tissue Regeneration and Organ Fibrosis (Wound Healing)

Type II EMT has a reparative role by producing fibroblasts and other related cells to reassemble the damaged and injured tissues [9] (Table 1). Accordingly, such classification of EMT mostly relates to wound healing, tissue regeneration, and organ fibrosis [2,18]. The wound healing process in cutaneous injuries occurs in three key steps: the initial inflammatory response; re-epithelialisation, which represents the regenerative stage; and extracellular matrix (ECM) remodelling, which is the final stage [18]. Under the inflammatory phase, neutrophils, monocytes, and macrophages release inflammatory factors such as IL-1, IL-6, and tumour necrosis factor (TNF)-a to get rid of the microbes and cellular debris at the wound site [19]. Type II EMT plays a key role in the generation of the fibrotic tissue where epithelial cells transform into profibrotic and pro-inflammatory myofibroblasts [20] that express mesenchymal markers like a-SMA and Vimentin [21]. During the EMT process, post-transcriptional regulatory machineries, including those mediated by microRNAs, regulate skin fibrosis, TGF-β signalling, fibroblast proliferation and differentiation, and finally ECM deposition [22]. TGF-β1 is also an important inducer of EMT in fibrosis [23]. Many of these factors involved in the induction of EMT comprise the pool of pathogenic factors in tissue fibrogenesis after tissue injury. Surprisingly, renal expression of TGF-β1 in diabetic nephropathy and poor renal functioning has been identified to be correlated. Therefore, anti-renal tubular interstitial fibrosis and EMT were caused by the inhibition of TGF-β1 [24]. Moreover, myofibroblasts are also known to cause tissue dysfunction and organ breakdown if uncontrolled [24].

### 2.3. Type III EMT: Associated with Cancer Progression and Metastasis

In cancer biology, EMT plays a very significant and complicated role in enhancing metastatic dissemination, whereby cancerous cells can leave their primary tumour locations and move to other areas and tissues [25]. The evolving understanding of the formation and development of epithelial carcinomas, particularly the mechanisms underlying tumour progression, has highlighted EMT as a crucial aspect in both in vitro and in vivo studies [5,25,26].

Unlike types I and II, type III EMT is specifically linked to the development of tumours by promoting the phenotypic change of epithelial cells into cancerous mesenchymal cells that are more aggressive, invasive, and capable of spreading [27,28,29] (Table 1 and Table 2). Additionally, type III EMT is crucial for tumour cells to avoid being eliminated by conventional cancer treatments and the immune system. This includes overcoming oncogene addiction and programmed cell death mechanisms like apoptosis, anoikis, and cellular senescence, all of which would otherwise impede tumour growth and survival [29,30]. To help cancer cells avoid immune surveillance and encourage extracellular matrix remodelling, EMT signalling and its constituents initiate immunosuppressive signals. This promotes tumour cell migration, invasion, and the formation of metastatic niches while also lowering host immune responses by dynamically remodelling the extracellular matrix, thereby reinforcing cancer cell metastatic potential through the structural and functional interplay between ECM and EMT [27,28].

The heterogeneous nature of tumours, including the presence of different tumour cell subtypes like cancer stem cells (CSCs), has been demonstrated in many studies to contribute significantly to the increased risk of relapse in cancer patients [31]. CSCs contribute to recurrence after initial treatment due to their increased capacity for survival and regeneration. Studies confirm that a high risk of developing and maintaining relapse-prone CSCs is linked to the process of ECM remodelling [31,32]. However, research on pan-cancer analyses of EMT models has shown that EMT signature genes differ significantly between cell populations and cancer types [31,33]. This variation makes it more challenging to develop universal EMT-targeted treatments, highlighting the need for more advanced and specialised approaches in each cancer type.

Prolonged inflammation and hypoxia, which are typical characteristics of fibrotic and malignant tissues and the tumour microenvironment, also serve as potent inducers of EMT (Table 3). In addition to encouraging EMT, these stressors also trigger transcriptional regulators and signalling pathways that aid in tumour growth, immunological evasion, and metastatic dissemination [34]. Collectively, all these observations support the idea that EMT is a complex, adaptive programme closely related to the characteristics of cancer, rather than just a cellular transition.

**Table 2:** EMT-associated markers and their involvement in diverse carcinomas.

Cancer Type	EMT-Related Factor	Role/Functional Insight	Ref.
Lung	EMT markers	Associated with advancement of disease	[35]
Colorectal	N-cadherin	Drives malignant transformation and tumour progression	[30]
Pancreatic	ZEB1	Promotes tumour growth, invasion, and metastasis in mouse models	[36]
Breast	Snail	Linked with invasive ductal carcinoma and nodal metastasis; expressed during carcinoma development	[37,38]
Colorectal	ZEB2	Acts as a prognostic biomarker; strongly expressed at invasive tumour fronts	[39]
Hepatocellular	TWIST1	EMT inducer supporting metastatic behaviour and invasiveness	[40]
Colorectal	SLUG	Strongly associated with tumour progression and poor clinical outcome	[41]
Bladder	Various EMT markers	Indicator of tumour stage and grade	[42]
Breast	Snail	EMT-promoting factor enhancing metastasis and tumour proliferation	[43]
Prostate	E- to N-cadherin switch	Hallmark EMT event strongly tied to cancer progression	[44]
Breast	HER2	Induces mammary tumours that spontaneously exhibit Snail expression and EMT phenotypes	[38]
Breast	TWIST1	Enhances mammary carcinoma development in mouse models	[45]
Bladder	E-cadherin	Shows inverse association with tumour grade and prognosis severity	[46]

**Table 3:** Disease conditions and associated EMT markers.

Conditions/Factors	EMT-Related Marker(s)	Functional Role	Ref
Kidney, liver, and intestinal inflammation	Discoidin receptor tyrosine kinase 2 (DDR2)	Marks epithelial cells undergoing EMT	[47,48]
Breast cancer	Fibroblast growth factor-10 (FGF-10)	Enhances cell viability, migration, colony formation, and wound repair; elevates mesenchymal markers; reduces apoptosis via increased GSK3β inhibition	[24,47,48]
Organ fibrosis	FSP1, α-SMA	Identifies mesenchymal phenotype acquired after EMT	[47,49]
Chronic inflammatory states	Cytokeratin, E-cadherin, FSP1, α-SMA	Detects epithelial cells transitioning through EMT	[24,49,50]

### 2.4. Partial or Hybrid EMT Phenotypes

The traditional paradigm of EMT had long been considered a binary switch: epithelial cells either switch completely to mesenchymal cells via EMT or revert to their epithelial state through MET [34]. This notion means that epithelial cells or mesenchymal cells are defined as identities that are distinct and mutually exclusive. This distinction, however, has been challenged by more recent studies indicating that EMT consists of many intermediate states and occurs as a continuum [27,51]. These intermediate states are also referred to as E-M hybrid, pseudo-EMT, or partial EMT and are defined by cells in a dynamic and plastic state that develop mesenchymal traits of invasiveness and motility while retaining some epithelial features [46]. It is important to note that these intermediate states are not just transient but are states with definite functional attributes.

A recent in vivo lineage-trace experiment based on high-resolution single-cell CRISPR barcoding technologies showed that the progression of EMT is not staged but instead manifested as a transcriptional continuum [51]. The late hybrid EMT cell types could produce a survival and metastatic advantage over the fully mesenchymal cell types because they selectively disseminate in metastatic cancer models [51]. The EMT pathway influences the expression levels of ECM genes, such as fibronectin and collagen isoforms [46,52]. EMT-related transcriptional programmes are often context-related and work along signalling cascades with examples like the cancer stem cell marker CD44 and Inhibin Beta A (INHBA) that codes for a TGF-β super family member protein. Studies suggest the expression of these two proteins to be upregulated, plateaued, or even repressed depending on the particular signalling pathways involved [47,49].

It is essential to mention that metabolic reprogramming, namely the transition from oxidative phosphorylation to glycolysis, correlates with the elevated activation of mTOR signalling in the EMT hybrid populations [49,52]. In the same light, EMT hybrid cells enhance the proliferation-related genes. In addition, ZEB1, ZEB2, SNAIL, and SLUG transcriptional factors are overexpressed in the EMT pseudo-state, which correlates with invasion and metastasis [49,52]. These all lead to tumour heterogeneity and therapeutic resistance by silencing the epithelial markers in addition to the activation of mesenchymal and stemness-related genes. Interestingly, it has also been shown that cells in late hybrid EMT states or closer to the mesenchymal end of the spectrum have a higher metastatic potential as compared to complete EMT states. The cells are essential in tumour growth and recurrence because they proliferate more, have a high metabolic flexibility, and activate pro-survival pathways [46,49,52].

Transcriptomics and computational modelling at the single-cell level have enhanced our knowledge of EMT plasticity. In a recent paper, the EMT states of various cancer types were classified using large language models that are trained on single-cell transcriptomic data [53]. This has defined distinct hybrid EMT niches with varied immune profiles that can proliferate. The findings emphasise the role played by spatial and temporal contexts in the EMT regulation and suggest the possibility of therapeutically modulating the hybrid EMT states to avoid metastasis without damaging healthy tissue [53]. The transition to a continuum-based model of EMT is an indication of a more profound understanding of cellular plasticity in cancer biology [49]. Intermediate EMT is in fact a distinct functional state that can lead to tumour invasion and metastasis, immune evasion, and treatment resistance [53]. Treatment approaches in the future may be improved by targeting regulatory networks that preserve these hybrid states including metabolic modulators, EMT-associated transcription factors, and phenotypic stability factors (PSFs).

Hybrid or partial EMT states are characterised by overlapping expression of epithelial markers (e.g., E-cadherin, EpCAM, cytokeratins), mesenchymal markers (e.g., N-cadherin, Vimentin), and the presence of stemness-related surface markers (e.g., CD44 high/CD24 low) [51]. The existence of these intermediate states causes tumours to become more aggressive. Late-hybrid EMTs have the greatest metastatic capability, and as a result of this, they can migrate as clusters of circulating tumour cells (CTCs), which are more resistant to apoptosis and 50 times more metastatic than single mesenchymal cells [54]. There are also greater stem-like characteristics, metabolic plasticity (i.e., the ability to freely switch between glycolysis and oxidative phosphorylation), immune evasion, and improved therapeutic resistance to chemotherapy, targeted therapies, and immunotherapy in hybrid EMT cells [55].

In spite of the fact that hybrid or partial EMT states are gaining recognition as having superior metastatic potential and therapeutic resistance compared to fully mesenchymal cells, the bulk of supporting evidence is based on single-cell transcriptomic and preclinical mouse models, but not large-scale human clinical datasets [51]. Hybrid phenotypes seem to be especially good for collective migration and stemness, but their frequency and functional significance vary a lot between different types of cancer and may be affected by the tumour microenvironment. It remains unclear whether these states are a universal driver of aggressiveness or are more prominent in specific cancer types such as breast, lung, and OSCC [56].

## 3. EMT-Inducing Signalling Pathways

Signalling pathways are a major inducer of the EMT programme [57,58]. As an example, EMT is induced by a variety of growth factors, such as transforming growth factor (TGF)-β, fibroblast growth factor (FGF)-β, hepatocyte growth factor (HGF), and epidermal growth factor (EGF), with intermediary signalling pathways such as Wnt, Sonic hedgehog (Shh), and Notch [58,59]. At the same time, cytokines like IL-8, IL-6, and tumour necrosis factor (TNF)-α are released in the tumour stroma, and work in concert to induce EMT within the microenvironment [58,59,60]. In addition, tumour cells interact with the ECM to induce and promote EMT [59].

### 3.1. TGF-β Induction

Transforming growth factor (TGF)-β signalling, being a strong inducer of EMT, is linked to tumour metastasis and progression, alterations of tumour immunity, and organ fibrosis [13]. TGF-β-induced EMT was also shown to be associated with the dynamic control of epigenetic markers, such as H3K27me3, H3K9me3, and DNA methylation, where H3K9me2 was found to decrease and H3K4me3/H3K3me3 to increase during the EMT induction in the hepatocyte cell line AML12 [60,61,62,63]. The modifications in the epigenetic marks are conditional on LSD1 lysine-specific Demethylase 1(LSD1) [62,63]. It was further shown that TGF-β-induced EMT, cell migration, and chemoresistance are mediated by the loss of LSD1 [63]. Remarkably, the influence of TGF-β as an EMT-stimulating mediator in breast cancer was also clear in epithelial PyMT-1099 cancer cells of MMTV-pyMT transgenic mouse models [52] where EMT was induced by treatment with the TGF-β cytokine, with the reverse process, MET, being triggered by cytokine withdrawal [52,64]. Further, the transcription factor YY1 has been linked to mediating TGF-β-induced EMT and profibrotic phenotype in A549 lung adenocarcinoma cells [21,23,24].

### 3.2. FGF Induction

Fibroblast growth factors (FGFs) are a group of proteins that bind to heparin and play vital roles in various biological processes, including cell differentiation, migration, angiogenesis, neurogenesis, and tissue repair [64]. FGF-β, initially found in the brain and pituitary gland, was one of the first FGFs identified in humans. Evidence suggests that FGF-β has a crucial impact on the development and progression of cancer and was shown to be significantly methylated in breast and colorectal cancer [65]. Moreover, Strutz et al. demonstrated that FGF-β can affect EMT since the growth factor was shown to downregulate the expression of cytokeratin in lung cancer cells [64]. FGF-β can also activate various signalling pathways, including the PI3K/Akt/mTOR and MAPK/ERK pathways, which are involved in FGF-β-induced E-cadherin downregulation and cell invasion in ovarian cancer cells [66]. Additionally, the DNA methylation-induced inactivation of SPRY2 (FGF receptor antagonist) can prevent cell proliferation, differentiation, and angiogenesis in endometrial cancer by inhibiting the RAS-MAPK pathway [67]. FGF-β can also interact with other growth factors to regulate EMT in cells, as demonstrated by Shirakihara et al., who showed that FGF-β and TGF-β1 induced synergistic effects during the induction of EMT in normal mammary epithelial cells [66].

### 3.3. HGF Induction

Hepatocyte growth factor (HGF) is a cytokine that is secreted by mesenchymal cells, and its transmembrane tyrosine kinase receptor is a heterodimer called MET, which is a product of the proto-oncogene c-MET expressed on epithelial cells [68,69]. When HGF binds to MET, it activates several signalling pathways, including MAPK and PI3K, that regulate different cellular functions such as proliferation, invasion, cell survival, branching morphogenesis, and migration of cancer cells [67,69]. It is shown that HGF inhibition alone can significantly reduce regional and distant metastasis in mice [70]. Moreover, HGF communicates via several intracellular signalling mechanisms, including the PI3K/Akt, ERK, COX-2, and Wnt pathways, in different cancer types, such as primary colon cancer [71,72]. During embryonic development, HGF-c-MET facilitates signalling between mesenchymal and epithelial cells. HGF signalling is also linked to promoting cancer progression through EMT. HGF phosphorylates β-catenin, disrupting cell–cell adhesion, which in turn results in the redistribution of E-cadherin to non-adhesion areas [69]. For instance, a study has suggested that high expression of HGF, activated by HGF promoter methylation, induced EMT, cell migration, and invasion in prostate cancer [73].

### 3.4. EGF Induction

Epidermal growth factor (EGF) plays a critical role in enhancing cell growth, proliferation, tumour invasion, and metastasis, and acts as a potent inducer of EMT in epithelial malignancies [74,75,76]. Aberrant expression of EGF and its binding site EGFR can directly or indirectly activate and regulate different downstream cancer-related signalling molecules, including PI3K/Akt, Wnt/β-Catenin and ERK/MAPK, all of which are capable of EMT induction [74,75,76]. The initiation of the downstream signalling pathways can result in the upregulation of EMT-related TFs and mesenchymal markers (Vimentin and N-cadherin) [77,78,79]. In breast cancer, downstream activation of ERK1/2-phospho-Smad2/3 in response to EGF promoted EMT by upregulating Snail and, consequently, suppressing E-cadherin expression [79,80,81,82,83]. The concept of EGF promoting EMT via PI3K/Akt and MEK/MEK signalling pathways was also demonstrated in salivary adenoid cystic carcinoma (SACC) [80]. The inhibition of EGFR downregulates both PI3K/Akt and MEK/ERK, suppressing SACC tumour proliferation, invasiveness, and migration [80]. Additionally, EGF can also cooperate with other factors in enhancing the induction of EMT in various carcinomas, including TGF-β in prostate cancer [78,81], FAK in colorectal cancer [82], and Src in gastric cancer [77].

### 3.5. Wnt Induction

The Wingless/Int-1 (Wnt) pathways consist of noncanonical (β-catenin-independent) and canonical (β-catenin-dependent) pathways [84,85]. The canonical Wnt, also referred to as Wnt/β-catenin, is activated by secreted Wnt ligands binding to their receptors (Fzd and LRP), involving the stabilisation and translocation of cytoplasmic β-catenin into the nucleus, which then acts as a transcriptional switch controlling multiple cellular processes, including cell proliferation [86]. The links between the Wnt signalling pathway, EMT, and tumourigenic behaviours have been demonstrated in a number of studies, where misregulated Wnt pathway activity is associated with enhanced tumour invasiveness and metastasis, a high recurrence rate, and poor prognosis in cancer patients [86]. In addition, upregulated Wnt level is also found to be associated with high resistance to anti-cancer therapies in OSCC [27,85] and lung cancer [86]. Wnt/β-catenin signalling has been aggressively implicated in different types of carcinomas as a key inducer of EMT, individually or synergistically with TGFβ in breast cancer [87] or with Notch in colon cancer [88].

### 3.6. Notch Induction

The Notch signalling pathway is involved in many crucial cellular processes of both healthy and cancerous cells, including cell proliferation, differentiation, apoptosis, and survival. Notch signalling is activated when the Notch ligand binds to an adjacent Notch transmembrane receptor of a neighbouring cell [89,90]. Dysregulation of Notch signalling has been studied as an oncogenic factor, suppressing cellular apoptosis and promoting cell survival, as well as invasive and metastatic mechanisms, including EMT [91,92]. An upregulation of Notch ligands and receptors was detected in oral squamous cell carcinoma, which was then demonstrated to correlate with enhanced EMT of the cancer cells [92,93,94]. Inhibition of Notch signalling resulted in downregulation of EMT-TF Snail and the mesenchymal marker Vimentin, along with upregulation of E-cadherin [92,93,94]. Notch signalling is also shown to be a vital factor for hypoxia-induced EMT in colorectal cancer, ovarian carcinoma, breast cancer and cholangiocarcinoma cells [89,90,91,94]. In this study, Notch was shown to be directly associated with the upregulation of Snail-1, a critical EMT-related TF [91]. In addition, the inhibition of Notch signalling reversed hypoxia-induced suppression of E-cadherin and restored the epithelial phenotype of the cells [95].

### 3.7. Shh Induction

The Sonic hedgehog (Shh) signalling pathway is crucial in maintaining stem cells, as well as in specifying cell growth patterns and differentiation during embryonic development [96,97]. It allows for the transfer of signals from the extracellular environment to activate gene expression involved in cell survival and proliferation [98]. The signalling cascade is activated when Shh binds to the receptor Patched (Ptch), initiating the activation of a downstream protein called Smoothened (Smo). Through this pathway, a family of transcription factors (Gli1, Gli2, and Gli3) becomes activated, which further induces the expression of their target genes [97]. Recent research has indicated that the Shh pathway is dysregulated in several types of cancer and can induce EMT individually or in cooperation with other pathways such as TGF-β, as observed in lymphatic and gastric tumours [96], pancreatic cancer [98], and oesophageal squamous cell carcinoma [99]. As an example, gastric cancer cells treated with Shh exhibited altered cell morphology consistent with EMT, as well as a strong suppression of E-cadherin and upregulation of Snail [96]. Moreover, it has been demonstrated that the physical binding of activated Gli1 and Gli2 to the promoter regions of specific genes, including oncogenes and those associated with the EMT process, such as Bmi1, Nanog, and Snail1, can directly facilitate their expression [97,98,99,100].

### 3.8. TNF-α Induction

Tumour necrosis factor (TNF)-α is a pro-inflammatory cytokine by which cancer invasion and metastasis are mediated [101,102]. TNF-α treatment in cell models of colorectal cancer led to an increase in the expression of fibronectin and N-cadherin in addition to EMT-inducing transcription factors such as Snail, Slug, ZEB1, and Twist, while reducing the levels of E-cadherin and the epithelial tight junctions, Zona occludens [103,104]. Further, treatment of HCT116 colorectal cancer cells with TNF-α induced the AKT pathway and suppressed the activity of GSK3, thus leading to overexpression of Snail [104]. The inhibition of GSK3ß activity is regulated by the P13K/AKT pathway, which allows for the phosphorylation of serine nine [102,103]. Conversely, TNF-α in renal cancer cells was found to activate several signalling pathways, such as E-cadherin inhibition, Vimentin upregulation, and metalloprotease 9, leading to EMT [103]. Models of head and neck squamous cell carcinoma indicate that TNF-α enhances the stabilisation of Slug through the induction of NF-kB activity and inhibition of Snail [101].

### 3.9. Hypoxia Induction

One of the microenvironmental factors that has been identified as having a high correlation in inducing cancer metastasis is hypoxia [105,106]. Hypoxic conditions stabilise hypoxia-inducible factor-1a (HIF-1a), leading to EMT induction and metastasis [50,105]. Misregulation of EMT transcriptional regulators, such as Snail, Twist1, ZEB1, ZEB2, and SIPI, has been implicated in hypoxia [50,60]. EMT markers are also controlled by chromatin modifiers under hypoxic conditions, such as histone deacetylase 3 (HDAC3), which controls the inhibition of epithelial genes while activating mesenchymal genes [58,61]. It has been shown that promoters of EMT in hypoxic conditions resonate between an active H3K4me and silenced H3K27 histone marks [58,61]. Ca^2+^ Signalling reduction due to intracellular calcium chelation in breast cancer is linked to EGF and hypoxia-induced EMT [74,105]. However, in lung cancer cells, exosome-mediated transfer of miR-193a-3p, miR-210-3p, and miR-5100 has been observed, theoretically suggesting that the miRNA plays a role in invading the cell through EMT signalling via STAT3 [106]. Thapsigargin, in A549 and H358 lung adenocarcinoma, suppresses the endoplasmic reticular Ca^2+^ ATPase, which leads to a large-scale downregulation of E-cadherin and upregulation of Vimentin [106].

### 3.10. Integrins

Integrins are a diverse family of transmembrane receptors that facilitate interactions between cells and the ECM. They function as dynamic signalling molecules that facilitate mechanical and biochemical communication from the tumour microenvironment (TME), influencing tumour growth, migration, and invasion, according to growing evidence of their role in the progression of cancer [107,108,109,110]. Several integrin subtypes, such as αvβ3, αvβ6, αvβ8, and β1, are essential for controlling EMT as tumours advance [101,102,103,104,105,106,107,108,109,110,111,112,113,114]. They were shown to be frequently elevated in aggressive tumours, initiating EMT by activating critical signalling pathways including TGF-β, Wnt/β-catenin, and PI3K/Akt [115]. In breast cancer, TGF-β increases FGF1 signalling and mesenchymal differentiation by stimulating mammary epithelial cells to produce αvβ3 [114].

Furthermore, integrin αvβ3 has been demonstrated to induce EMT-like phenotypic changes in glioblastoma, via the induction of the PI3K/AKT signalling pathway, hence increasing tumour invasiveness and mobility [116,117]. These findings highlight the role of integrins as signal amplifiers that initiate EMT, a crucial aspect in tumour metastasis.

Poyyakkara et al. emphasise the intricate role of integrin β4 in activating EMT via the ITGB4–AKT–miR-383–GATA6 pathway [117]. It shows that while miR-383 inhibits GATA6 to reverse EMT, ITGB4 activates AKT and β-catenin signalling [117]. Similarly, integrin α6/CD49f is associated with the epithelial–mesenchymal cooperation (EMC), particularly in muscle-invasive cancers, where it interacts with growth factor receptors and E-cadherin to promote tumour progression across laminin-rich barriers [118,119,120,121]. In addition to promoting transcriptional reprogramming via SMAD, these integrins have been linked to enhanced invasiveness and a poor prognosis [122,123]. Interactions of integrin β1–fibronectin stimulate FAK/Src signalling, which encourages EMT and metastasis, but α6β4 mostly promotes EMT through Rac1 and NF-κB signalling, especially in triple-negative breast cancer subtypes [114,124]. Moreover, integrin β1 is upregulated in EMT, as it activates integrin-linked kinase (ILK), which increases Snail levels and reduces E-cadherin [125].

When cancer cells are in the state of mesenchymal transition, they secrete matrix metalloproteinases (MMPs) and other remodelling enzymes that degrade and remodel the surrounding matrix [126]. This altered ECM, highly enriched in fibronectin, collagen, and laminin, also provides new ligands for integrin binding, further increasing their migratory and invasive properties. This underscores the interactions of integrin with the ECM that promote cancer cell migration and maintenance of EMT through the constant activation of survival and motility pathways [111]. The association between integrin-mediated EMT, enhanced stem-like characteristics and response to therapy gave a therapeutic angle to integrin-mediated EMT in clinical cancer research [112]. Despite the encouraging results of integrin targeting in preclinical models, there is still no way to apply the results in clinical practice because of tumour compensatory and functional redundancy.

### 3.11. Matrix Metalloproteinases (MMPs)

Matrix metalloproteinases (MMPs) are a family of ECM-degrading endopeptidases that have gained attention as factors involved in promoting EMT as well as in the regulation of ECM remodelling. The mechanistic relationship that exists between MMPs and EMT of carcinoma cells has been analysed in many studies [126,127,128]. In addition to their established role in degrading the extracellular matrix by acting as zinc-dependent enzymes, MMPs are important in signalling pathways that regulate EMT-controlling transcription factors and support EMT-mediated migration of carcinoma cells. It is important to note that MMP3 was also identified to activate EMT by causing reactive oxygen species (ROS) production and activating Rac1b, which, in turn, activates Snail and compromises the integrity of epithelial cells [129]. Moreover, mechanistic studies have revealed MMPs to be necessary in initiating several cellular processes. For example, MMP9 facilitates the process of cellular migration and detachment through the upregulation of Snail and E-cadherin cleavage that facilitates EMT [130]. The relationship between MMPs and EMT-TFs is two-way: ZEB1 elevates the expression of MMP2 in non-small-cell lung cancer, whereas Twist1 stimulates the activity of MMP7 in oesophageal squamous cell carcinoma to induce phenotypic remodelling and resistance to chemotherapy [131,132]. This interaction is enhanced by hypoxia that stabilises HIF-1alpha signalling under low-oxygen conditions, enhancing the level of MMP and EMT-TF expression, which is conducive to angiogenesis and invasion [133,134,135]. This positive feedback loop is also observed in glioblastoma, where it has been established that the inhibition of MMPs turns back EMT-like features, and it also diminishes the aggressiveness of the tumour cell [136].

In other studies, MMP7 and MMP14 are observed to control TGF-β2, Wnt/β-catenin, and NF-κB pathways to maintain EMT and tumour metastasis [126,137]. High EMT marker expression and poor prognosis are linked with high MMP expression in numerous types of cancers, including colorectal, pancreatic, liver, and glioblastoma [128,136,137,138]. MMPs are important in tumour development, which is closely coupled with hypoxia and several signalling pathways. Despite their therapeutic potential, MMPs are hard to target because of their redundancy and context-specific effects. Nevertheless, due to their ability to enhance tumour sensitivity to chemotherapy and to reduce EMT marker expression, while re-establishing the epithelial phenotype, they are potential candidates for anti-metastatic therapies. Achieving such modulation of the MMP-EMT axis and preserving normal tissue remodelling requires that future studies be directed to the development of specific context-directed inhibition methods and biomarker strategies.

## 4. Cellular Glucose Metabolism and the Association Between Hyperglycaemia and Cancer Progression

### 4.1. The Production of Cellular Energy and Glucose Metabolism

**Figure 2:**
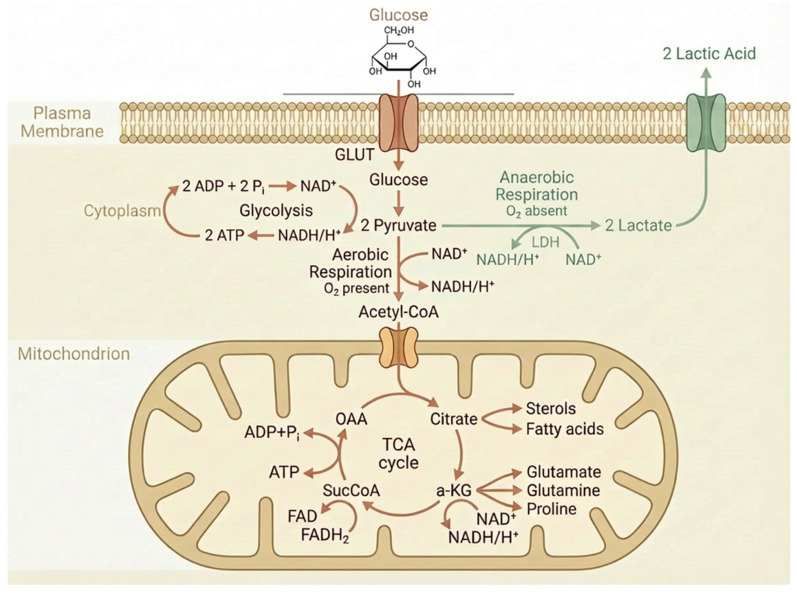
**Cellular respiration, glycolysis, and the TCA cycle.** Red arrows indicate aerobic respiration in oxygen-rich conditions. Blue arrows denote anaerobic respiration taking place in an oxygen-deprived environment. Key abbreviations: ADP = adenosine diphosphate; ATP = adenosine triphosphate; ETC = electron transport chain; FAD = flavin adenine dinucleotide; GLUT = glucose transporter; NAD = nicotinamide adenine dinucleotide; OAA = Oxaloacetate; TCA = Tricarboxylic Acid Cycle (created by GPAI).

Glucose is the major energy source in both cancerous and normal cells, and the metabolic process referred to as cellular respiration converts this molecule to adenosine triphosphate (ATP) and other forms of biomolecules that are essential for the survival and growth of the cells. It is through the glycolytic breakdown of glucose molecules that cells produce ATP and NADH, which are important constituents of cellular balance to both normal and cancerous populations [139,140,141]. The oxygen concentration of the cellular environment has a tremendous role in determining the metabolic pathway the glucose follows. Anaerobic metabolic pathways are used when the oxygen level is low, and the cells generate ATP. Under hypoxic conditions, lactate dehydrogenase sustains glycolysis by transforming pyruvate to lactate and restoring NAD+ [140,141,142]. Besides avoiding the entry of pyruvate into the oxidative metabolites in the mitochondrion, this oxygen radical-independent metabolite process also ensures continuous energy generation. On the contrary, glucose is catabolized through aerobic mechanisms when oxygen is readily accessible. Following entering the mitochondria, pyruvate is converted to acetyl-CoA and then goes through the citric acid cycle. This oxidative process generates substantial amounts of ATP, NADH, and FADH2, while releasing carbon dioxide as a waste product (Figure 2).

Through the electron transport chain, these reducing equivalents utilise oxygen as the terminal electron acceptor, thereby promoting effective ATP synthesis [142,143,144,145,146]. Aerobic glucose metabolism enables fast energy mobilisation, remarkably producing essential intermediate compounds for biosynthesis and cellular development at rates 10–100 times higher than full oxidative metabolism [142,143,144].

### 4.2. The Metabolic Reprogramming Linked to Cancer

Metabolic adaptability is a key feature of cancer biology, as cancer cells exhibit remarkable adaptability by switching from the usual aerobic breakdown of glucose to the use of various nutrient sources. The unique metabolic changes cancer cells exhibit when exposed to harsh environmental stressors enable unchecked growth and improved survival. This metabolic restructuring satisfies the raised requirements of energy and biomolecules to be able to survive and grow long-term in difficult tumour microenvironment conditions [143].

One of the main phenomena in cancer metabolism is the Warburg effect, in which glucose in cancer cells is transformed into lactate even when the organism has enough oxygen. This special modification of the cell respiration consists of a shift in glucose metabolism and the re-arrangement of lipid and amino acid metabolism [141,142,143,144]. The pathway facilitates tumour growth and progression, promotes its survival in adverse conditions, and meets the biosynthetic needs of fast-dividing cells, despite being less energy-efficient than oxidative processes.

To increase glucose uptake and fulfil the high energy requirements of the mitochondria, one way is to upregulate transport proteins, particularly GLUT-1, to compensate for the loss of ATP generation [141,144,145]. Although some primary malignancies have been reported to prefer the glycolytic metabolism in the production of ATP, other cancerous cell populations have been reported to show increased respiratory activity, with the implication that the Warburg effect does not necessarily indicate dysfunction of the mitochondrial compartment [146,147].

Nevertheless, as metastatic tumour cells have higher energy requirements, the consumption of mitochondrial respiration and oxidative phosphorylation in facilitating cellular motility and tissue infiltration is frequently increased in response to mitochondrial hypoxia. As metastasis progresses, the synthesis of macromolecular components of new cellular structures is necessary, leading to a shift to oxidative phosphorylation (OXPHOS) [145,146,147].

### 4.3. Clinical Importance of Glucose Regulation in Cancer Treatment

**Figure 3:**
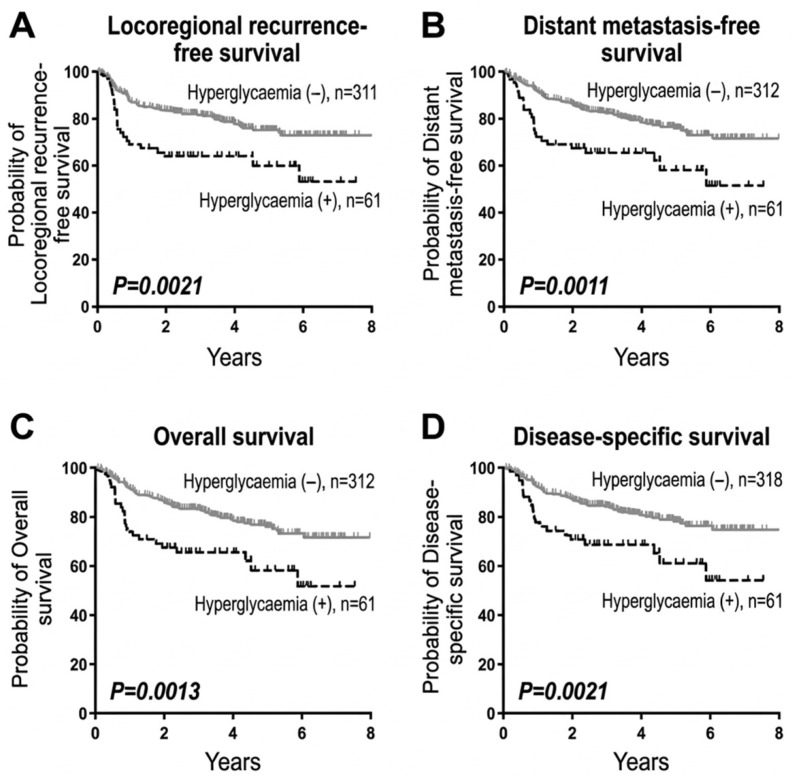
**Survival outcomes over five years for OSCC patients, comparing those with hyperglycaemia to those without.** The red line indicates patients with hyperglycaemia, while the blue line shows patients without hyperglycaemia. (**A**) Locoregional recurrence-free survival. (**B**) Distant metastasis-free survival. (**C**) Overall survival. (**D**) Disease-specific survival (figure reproduced from original data in [150] and by written permission from Dr Kai-Ping Chang).

Managing elevated blood glucose levels is vital for cancer patients to achieve the most effective possible treatment outcomes. Research has consistently shown a link between long-term high blood sugar levels in people with diabetes and a higher risk of developing various types of cancer, highlighting the complex relationship between diabetes, high blood sugar, and cancer risk [148,149,150,151,152] (Figure 3).

There is strong clinical evidence that hyperglycaemia is linked to poor cancer outcomes and possible EMT-driven resistance in oral squamous cell carcinoma (OSCC). Indicatively, elevated glucose is associated with higher tumour stage, longer hospital stays, poor response to treatment, higher local recurrence, and distant metastasis [150,151,152,153,154]. Diabetic patients are 1 to 41 times more likely to develop oral cancer than those without diabetes [154]. Additionally, diabetic patients with oral cancer have poorer survival rates compared to those without diabetes, according to mortality statistics. High glucose levels in patients with OSCC are strongly linked to more advanced tumour staging [151,152,153,154]. Patients with high glucose levels typically require more extended hospital stays compared to those with normal glucose levels. This increase in glucose levels is tied to reduced treatment effectiveness, poorer survival outcomes, and higher rates of local tumour recurrence and distant metastatic spread [151,152,153,154]. These findings indicate that metabolic stress can contribute to the aggressiveness of the tumour in certain types of cancer, although these observations are yet to be validated in a wider selection of cancer types. Direct mechanistic evidence to link high blood glucose with EMT-mediated therapeutic resistance is also yet to be established in more clinical models.

### 4.4. Species of Reactive Oxygen (ROS) and Hyperglycaemic Cell Stress

Increased oscillation of reactive oxygen species (ROS) could be used to increase glucose processing by mitochondria in the event of high blood glucose, where high concentrations of ROS-generating enzymes, such as NADPH oxidase and electron transport chain (ETC) components, are active. The increased levels of ROS and the impaired balance between the oxidative species and the antioxidant detoxification system led to oxidative stress. Hyperglycaemia-induced high oxygen consumption [155,156] can further result in the establishment of hypoxic pockets in the tumour microenvironment [155,156].

Elevated ROS concentration can have several adverse effects in the tumour tissues. ROS causes destruction of the genetic components and proteins of the cell and the lipids, as signalling molecules, and could cause genomic instability and genetic mutations through various growth factor signalling, cellular proliferation, survival pathways, and hypoxic adaptation responses. ROS can also induce anti-cancer signalling cascades by damaging cell components through oxidation and inhibiting the cell cycle, and this can limit tumour development and induce cancer cell death [156,157].

Despite the established knowledge that hyperglycaemia is capable of modifying radiation resistance and changing the metabolism of tumour cells, the mechanism behind this effect is not yet understood. Further research on the downstream effects of high blood glucose, including the molecular pathways involved, changes in metabolic by-products, and the link to cancer cell behaviour, therapy response, and metastasis, will shed light on the links and possible therapeutic avenues in this domain [158,159].

### 4.5. Links Between Epithelial–Mesenchymal Transition and Glucose Metabolism in Cancer

The metabolic capacity of epithelial carcinoma cells fuels the EMT process. In fact, cancer cells have the ability to modify their metabolism along phenotypic switches while still able to provide enough energy to other vital (cancer-related) processes. It has been shown that EMT is directly associated with altered expression of major glycolytic enzymes, suggesting that glucose metabolism may be correlated with this critical phenotypic transition [160,161,162]. These alterations influence the invasive property and metastatic ability of tumour populations by modifying modes and levels of glucose consumption [163]. These data suggest that a complex network of glucose processing pathways and EMT machinery regulates cancer cell behaviour and metastasis.

One key factor driving neoplastic remodelling is changes in how cells produce energy [164]. The aggressiveness of tumours is heavily influenced by metabolic processes that promote a more glycolytic profile [164]. As a result, tumours need to produce more glycolytic enzymes to initiate glycolysis [164]. In fact, the Warburg effect is a target for anti-cancer therapy [164]. These metabolic changes also help cancer cells transform into cancer stem cell-like entities that express transcription factors, triggering the epithelial–mesenchymal transition [164].

Reactive oxygen species (ROS) can form when high blood sugar levels create an environment with excessive oxidative stress and inadequate oxygen. These factors can further influence the epithelial-to-mesenchymal transition (EMT). If cells cannot regulate ROS, an oxidative burden can trigger a chain of signalling events that alter the cell identity. EMT is worsened in oxygen-deprived environments because hypoxia-responsive transcription factors stabilise and activate genetic programmes that support mesenchymal transformation. An imbalance between ROS production and antioxidant capacity leads to an oxidative burden that actively drives EMT through interconnected signalling networks [163].

Studies have shown that hypoxia and extreme glucose deprivation may induce the expression of Snail/Slug and N-cadherin [164]. The study of hypoxic conditions in the hepatocellular carcinoma cell line, HePG2, implies that hypoxia induces EMT-related proteins while reducing cellular proliferation [164,165,166]. Moreover, the phosphorylation of heat shock transcription factor (HSF) is essential for the regulation of SNF1-dependent glucose metabolism. Under severe hypoxia, NADPH is elevated in cancer cells to control the entry of glucose into the pentose phosphate pathway [165]. Interestingly, it has been discovered that Snail inhibits phosphofructokinase 1 (PFK-1), which favours metabolic reprogramming of the pentose phosphate pathway and glycolysis in metabolic demand, and thus enables cancer progression in conditions of oxidative demand [166]. The increased frequency of production of ROS under high-glucose conditions leads to the activation of oxygen-sensitive transcription factors that induce EMT [167]. This metabolic imbalance triggers a vicious cycle where the absence of oxygen accelerates the EMT process, making cells more mobile and resulting in the development of more aggressive and invasive tumours. The complex molecular relationships between oxidative stress, glucose upregulation, oxygen deprivation, and EMT processes influence the evolution of cancer and metastatic behaviour [167,168,169,170,171,172].

Research on clinical observations suggests that the body produces more hydrogen peroxide (H_2_O_2_) in response to stress caused by elevated blood sugar levels. An increase in H_2_O_2_ is directly associated with increased EMT activity and a higher risk of metastasis in pancreatic cancers, making it a marker and a crucial participant in the process that drives metastasis [167].

Through metabolic switching, glucose starvation can prevent TGF-β1-induced EMT and migration of hepatocellular carcinoma cells [168]. Research suggests that HSF1 inhibits EMT-associated migration through glucose starvation in a Snail-dependent way [171]. On the other hand, chronic glucose insufficiency promotes a mesenchymal phenotype and metabolic plasticity in ovarian carcinoma through a ZEB1/nicotinamide N-methyltransferase (NNMT) axis. In a glucose-independent process, NNMT plays a crucial role in ZEB1-induced mesenchymal gene expression [172]. When hepatocellular carcinoma cells were treated with metformin in a glucose-deficient environment, they showed an anti-migratory effect [171].

High glucose levels promote EMT, driven by Snail, and trigger the migration, invasion, and proliferation of gastric cancer cells by increasing ENO1 expression [169]. Elevated blood sugar levels enhance cancer cells’ ability to invade via caveolin-1-dependent pathways. This facilitates loss of E-cadherin and an increase in mesenchymal markers, which, in turn, leads to higher Slug mRNA levels in hormone-receptor-positive breast cancers [170]. Meanwhile, bladder cancers utilise modulation of the YAP1/TAZ pathway, and uterine cancers utilise the ER/GLUT4-mediated VEGF secretion pathway, highlighting the versatility of EMT activation strategies across different cancer types [171].

One of the major factors that determines the ability of TGF-β to trigger the EMT programme is the changes in metabolites (mitochondrial lipid choline and glycolysis) and reprogramming [173]. Although TGF-β suppresses the cyclin-dependent kinases or the G1 phase of the cell cycle as well as down-regulating pro-oncogenic transcription factors, the inhibition of cell proliferation and induced apoptosis of premalignant cells during disease progression is also a tumour-suppressing factor [173,174]. Nevertheless, at the advanced tumour stages, TGF-β becomes a promoter, which provides and facilitates the growth of cancer and malignancy [175,176,177].

To trigger angiogenesis, immune evasion, and EMT, cells in the carcinoma tumour microenvironment induce enhanced release of TGF-β, which in turn upregulates the expression of Glucose transporter 1 (GLUT1) as evidenced in breast cancer, glomerular and mesangial cells [178,179,180,181,182]. In fact, heightened expression of GLUT1 has been linked to TGF-β-induced EMT in breast cancer [179,181]. Increased levels of TGF-β result in the expression of hexokinase-2, which plays a vital role in glycolysis and the production of lactate, eventually resulting in metastasis [182,183]. There is other evidence that TGF-β2 inhibitors are radiation-sensitising and hence suppress therapy resistance [184,185,186]. An example is phosphodiesterase 4 (PDE4), an enzyme that transforms cAMP to AMP, which was shown to increase in TGF-β-induced EMT [184]. PDE4-targeted inhibitors enhanced MET markers in lung carcinoma cells and prevented the SMAD-independent TGF-β-induced EMT [184].

Even though the ROS-mediated activation of the TGF-β/PI3K/Akt/mTOR and MAPK pathway offers a mechanistic relationship between hyperglycaemia and epithelial–mesenchymal transition (EMT), most of the evidence is obtained under in vitro high-glucose-induced conditions or animal models of diabetes. These effects appear to be more intense in certain cancers, such as pancreatic, gastric, and OSCC, but little information exists on other types of tumours. Redundancy of pathways and context-specific signalling continues to complicate therapeutic targeting, and more stringent clinical trials will be required to determine causality [169,187,188,189] (Table 4).

**Figure 4:**
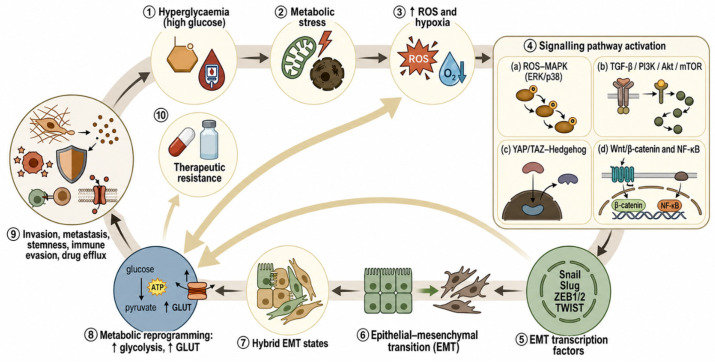
**Schematic diagram demonstrating the links between hyperglycaemic-induced metabolic stress, EMT and therapeutic resistance** (created by GPAI).

As discussed, hyperglycaemia can activate EMT in a variety of cancer types. However, developing customised treatment strategies to specific molecular contexts is crucial. Also, given that metabolic dysregulation is associated with phenotypic plasticity and the emergence of resistance to therapy, this emphasises the significance of closely monitoring glucose levels during cancer treatment. Notably, it is a two-way relationship in the form of a deleterious feed-forward cycle. Active reprogramming of glucose metabolism by EMT itself is essential to sustain the energy requirements of invasion and stress survival. Core EMT transcription factors directly tune metabolic genes; i.e., GLUT3 is transcriptionally activated by ZEB1 to promote glucose uptake and glycolysis in mesenchymal cells; phosphofructokinase PFKP (or FBP1) is repressed by Snail to increase the glucose flux into the pentose phosphate pathway to generate NADPH and maintain redox homeostasis [186]. All these EMT-induced metabolic phenotypes result in a stronger mesenchymal phenotype, as well as metabolic plasticity in the hybrid EMT phenotypes, and facilitate the excretion of drugs out of the body and resistance to therapy [165]. Hyperglycaemia therefore accelerates EMT, and EMT maintains glycolysis and elevated glucose dependence, which makes resistance self-reinforcing [185,186] (Figure 4).

Although this feed-forward metabolic–EMT loop has much preclinical evidence, there are still a few issues, which result in the necessity to be cautious. The majority of the research uses acute hyperglycaemic states rather than the chronic exposure of diabetic patients, and direct demonstration of improved glycaemic control and reduced EMT-mediated resistance in clinical outcomes is sparse and largely correlational. The heterogeneity of cancer types and the particular involvement of hybrid epithelial–mesenchymal transition (EMT) states in treatment failure in people with hyperglycaemia are critical gaps to be considered in the future, which require interventional research [165,180].

The best clinical links between hyperglycaemia and adverse outcomes (including possible EMT-related progression) are reported in OSCC and in pancreatic cancer, and preclinical and observational evidence in support of hyperglycaemia-induced matrix-specific EMT and chemoresistance is available in breast cancer [170,190]. Nevertheless, it remains to be seen through prospective validation whether better glycaemic control can significantly lower EMT-based resistance in the context of different cancers.

## 5. Conclusions

The phenotypic shift of epithelial cells along the E-M axis, also known as epithelial–mesenchymal transition (EMT), is a complex, multifaceted biological process rather than a simple change in cellular structure and components. According to multiple studies, EMT is an essential mechanism regulating key cell physiological and pathological processes, the progression of epithelial carcinomas, and the development of resistance to anti-cancer therapies. EMT is typically classified into three types based on the biological functions and processes it participates in: type I during normal embryogenesis; type II, linked to fibrosis and wound healing; and type III, which promotes malignant transformation, tumour local invasion, and distal metastasis [1,2,4,8,9,10,18,25,26].

Studies across various cancers have implicated the clinical significance of EMT-related markers as therapeutic targets for their association with poor clinical outcomes [33,34,35,36,37,38,39,40,41,42,43,44]. However, efforts to translate these markers into practical applications and to develop a universal biomarker are hampered by variation in EMT genetic signatures across carcinomas, suggesting that cancer-specific EMT profiles will be required for precise cancer prognosis and treatment strategies [28,29,30,31].

The emerging discovery of populations of carcinoma cells existing in the intermediate states, exhibiting high levels of plasticity and both epithelial and mesenchymal traits, characterised as hybrid, partial, or intermediate EMT, has challenged the conventional binary view of EMT [27,45,46,51]. Notably, the fact that late EMT states might have higher invasive and evasive potential than complete mesenchymal conversion suggests that therapeutic strategies should account for the spectrum of cellular plasticity along the EMT axis and the intermediary states [51]. The overlapping transcriptional networks involving core EMT transcription factors (Snail, TWIST, SLUG, and ZEB) are activated and regulated by multiple growth factors (TGF-β, FGF, HGF, EGF) and signalling pathways (Wnt, Notch, Shh), integrating with the signals from inflammatory mediators (TNF-α) and environmental stressors (hypoxia) [4,5,6,7,59,60,61,62,63,64,65,66,67,68,69,70,71,72,73,74,75,76,77,78,79,80,81,82,83,84,85,86,87,88,89,90,91,92,93,94,95,96,97,98,99,100,101,102,103,104,105,106,107]. This convergence underscores the evolutionary role of EMT as a basic adaptive mechanism that can be triggered by diverse stimuli to guarantee cellular survival.

The intricate relationship of integrins (ITGs) and matrix metalloproteinases (MMPs) during EMT regulation demonstrates how biochemical and mechanical stimuli interact during cellular phenotypic transformation [110,111,112,113,114,115,116,117,118,119,120,121,122,123,124,125,126,127,128,129,130,131,132,133,134,135,136,137,138]. During EMT, MMPs and EMT transcription factors interact continuously in self-reinforcing loops to sustain the mesenchymal phenotype while reshaping the ECM to facilitate invasiveness and tumour metastasis. In addition, beyond phenotypic alterations, EMT has been shown to be strongly associated with cellular glucose metabolism, with significant implications for the development of therapeutic resistance [142,143,144,145,146,147,148,149,150,151,152,153,154,155,156,157,158,159,160,161,162,163,164,165,166,167,168,169,170,171,172,173,174,175,176,177,178,179,180,181,182,183,184,185,186,187,188,189,190]. The regulatory axis comprising metabolism and EMT facilitates metabolic plasticity in cancer cells, creating intermediates to stabilise transcription factors, and powers the energy-intensive process of cellular transformation, which altogether enables these cells to overcome the damaging effects of cancer treatment [157,158,159,160,161,162,163,164,165,166,167,168,169,170,171,172,173,174,175,176,177,178,179,180,183].

Studies on YAP1/TAZ in bladder cancer and ER/GLUT4-mediated mechanisms in uterine cancer are two examples of how different carcinomas utilise different metabolic pathways to support EMT, indicating a temporal and context-dependent aspect among the two processes [171]. A study on the correlation between glucose metabolism and EMT in breast cancer found that GLUT1 expression is suppressed during acute TGF-β-induced EMT but is restored after prolonged exposure, allowing cells to maintain invasive and therapy-evasive traits without growth inhibition [178,179,180,181,182]. Also, EMT-TFs, including TWIST, have the ability to activate several glucose transporters (GLUT1, GLUT3, and GLUT12), which improves glucose uptake, creating a feed-forward loop that amplifies both metabolic and phenotypic features of EMT [178,179,180,181,182] . Besides being able to address biosynthetic demand, the elevated glucose uptake in EMT-transformed cells can contribute to increasing drug resistance by reinforcing the efflux pathways, suggesting that interference with the glucose metabolism may target EMT at its core and therefore offer a potential method of overcoming treatment resistance [184,185,186].

Intermediate EMT states are identified to have unique metabolic profiles that sustain the survival of cancer cells during therapeutic stressors through metabolic plasticity that alternates between glycolysis and oxidative phosphorylation according to nutrient availability and treatment pressure [160,161,162,163,164,165,166,167,168,169,170,171,172,173,174,175,176,177,178,179,180,183]. Proliferative signalling is synchronised with metabolic reprogramming through the mTOR signalling pathway, making chemotherapy and targeted therapy more resilient. This implies the need for therapeutic intervention that targets the entire spectrum of EMT states rather than treating EMT as a binary process, especially in light of evidence that late hybrid states have higher metastatic potential than fully mesenchymal cells [27,47,48,49].

Therapeutic approaches are complicated by the fact that metabolic stress itself plays vital roles in initiating EMT and in stabilising hybrid states, hence creating a paradoxical situation in which metabolism-targeted therapies may inadvertently promote the phenotypic plasticity they are intended to prevent. EMT induction and therapy resistance are promoted concurrently in hyperglycaemic conditions, which are prevalent in cancer patients. Higher recurrence rates, poor treatment responses, and more aggressive tumours are all associated with elevated glucose levels [148,149,150,151,152,153,154]. These clinical findings are mechanistically supported by ROS-mediated activation of EMT-TFs and metabolic conditions, including hyperglycaemia [155,156,157,158,159]. EMT induced by TGF-β enhances the levels of glycolytic enzymes and glucose transporters, generating metabolites, which strengthen the resistance and stabilise the mesenchymal state, establishing a feed-forward loop between TGF-β signalling and glucose metabolism [178,179,180,181,182].

Although the interplay between hyperglycaemia-induced metabolic stress and EMT offers an attractive mechanistic framework, caution is warranted when considering clinical translation. While preclinical evidence strongly supports the biological plausibility of this axis, direct clinical evidence that strict glycaemic management can reduce EMT-driven therapeutic resistance remains limited and largely correlative. Glycaemic control may therefore represent a low-risk, feasible adjunctive approach worth investigating in future prospective trials, particularly in hyperglycaemic patients with OSCC or pancreatic cancer. However, it cannot yet be recommended as standard clinical practice based on the current literature.

The mutual interaction of EMT and glucose metabolism opens new possibilities for therapeutic intervention. However, a clearer understanding of the connection between metabolism and EMT, cautious design of therapies, and cancer-specific molecular and metabolic profiling are required for efficient clinical outcomes.

## Figures and Tables

**Table 1:** Summary of EMT classification.

Classification of EMT	Function	Key Markers	Ref.
Type I	Implantation Embryogenesis Morphogenesis/Organ development	Plays a role during embryogenesis in differentiation into diverse cell types in sequential EMT and MET processes. Leads to no fibrotic or malignant transformation.	[1,2,4,8,9,10]
Type II	Organ fibrosis Tissue regeneration Wound healing	As a result of trauma and inflammatory injury; role in formation of fibroblasts in tissue regeneration. Involved in fibrosis with minimal effect in invasive capacity.	[1,9,10]
Type III	Cancer initiation, progression and metastasis	Role in generating cancer cells, contribution in tumour metastasis and acquired resistance to treatment. Pro-invasive and pro-metastatic impact on cells.	[1,2,3,4,5,6,7,8,9,10]

**Table 4:** Signalling pathways linking hyperglycaemia-induced metabolic stress to EMT.

Pathway	Key Mechanism	Main EMT Outcome	References
ROS → MAPK (ERK/p38)	ROS from high glucose activates ERK/p38	Snail/Slug upregulation, E-cadherin loss	[187,188]
TGF-β/PI3K/Akt/mTOR	ROS enhances TGF-β → PI3K/Akt/mTOR activation	Snail stabilisation, mesenchymal markers	[189]
YAP/TAZ-Hedgehog	Glycolytic reprogramming activates YAP/TAZ	EMT-TF induction, invasion	[169,189]
Wnt/β-catenin and NF-κB	High glucose activates Wnt and NF-κB	Snail/ZEB upregulation, chemoresistance	[188]

## Data Availability

No new data were created or analyzed in this study. Data sharing is not applicable.

## Abbreviations

The following abbreviations are used in this manuscript:

EMT	Epithelial-to-Mesenchymal Transition
MET	Mesenchymal-to-Epithelial Transition
FGF	Fibroblast Growth Factor
iPSC	Induced pluripotent stem cell
ECM	Extracellular matrix
IL	Interleukin
TNF	Tumour Necrosis Factor
CSC	Cancer stem cell
CTC	Circulating tumour cell
PSF	Phenotypic stability factor
FSP	Fibroblast-specific protein
HGF	Hepatocyte growth factor
EGF	Epidermal growth factor
Shh	Sonic hedgehog
SACC	Salivary adenoid cystic carcinoma
OSCC	Oral squamous cell carcinoma
TGF	Transforming Growth Factor

## References

[1] Lee J.M., Dedhar S., Kalluri R., Thompson E.W. (2006). The Epithelial-Mesenchymal Transition: New Insights in Signaling, Development, and Disease. J. Cell Biol..

[2] Thiery J.P., Acloque H., Huang R.Y.J., Nieto M.A. (2009). Epithelial-Mesenchymal Transitions in Development and Disease. Cell.

[3] Yang J., Weinberg R.A. (2008). Epithelial-Mesenchymal Transition: At the Crossroads of Development and Tumor Metastasis. Dev. Cell.

[4] Acloque H., Adams M.S., Fishwick K., Bronner-Fraser M., Nieto M.A. (2009). Epithelial-Mesenchymal Transitions: The Importance of Changing Cell State in Development and Disease. J. Clin. Investig..

[5] Tam W.L., Weinberg R.A. (2013). The Epigenetics of Epithelial-Mesenchymal Plasticity in Cancer. Nat. Med..

[6] Valastyan S., Weinberg R.A. (2011). Tumor Metastasis: Molecular Insights and Evolving Paradigms. Cell.

[7] Brabletz T. (2012). To Differentiate or Not-Routes towards Metastasis. Nat. Rev. Cancer.

[8] Hay E.D. (2005). The Mesenchymal Cell, Its Role in the Embryo, and the Remarkable Signaling Mechanisms That Create It. Dev. Dyn..

[9] Kalluri R., Weinberg R.A. (2009). The Basics of Epithelial-Mesenchymal Transition. J. Clin. Investig..

[10] Lim J., Thiery J.P. (2012). Epithelial-Mesenchymal Transitions: Insights from Development. Development.

[11] Buckingham M., Bajard L., Chang T., Daubas P., Hadchouel J., Meilhac S., Montarras D., Rocancourt D., Relaix F. (2003). The Formation of Skeletal Muscle: From Somite to Limb. J. Anat..

[12] Nieto M.A. (2013). Epithelial Plasticity: A Common Theme in Embryonic and Cancer Cells. Science.

[13] Lamouille S., Xu J., Derynck R. (2014). Molecular Mechanisms of Epithelial-Mesenchymal Transition. Nat. Rev. Mol. Cell Biol..

[14] Eastham A.M., Spencer H., Soncin F., Ritson S., Merry C.L.R., Stern P.L., Ward C.M. (2007). Epithelial-Mesenchymal Transition Events during Human Embryonic Stem Cell Differentiation. Cancer Res..

[15] Mercado-Pimentel M.E., Runyan R.B. (2007). Multiple Transforming Growth Factor-β Isoforms and Receptors Function during Epithelial-Mesenchymal Cell Transformation in the Embryonic Heart. Cells Tissues Organs.

[16] Fischer A., Steidl C., Wagner T.U., Lang E., Jakob P.M., Friedl P., Knobeloch K.P., Gessler M. (2007). Combined Loss of Hey1 and HeyL Causes Congenital Heart Defects Because of Impaired Epithelial to Mesenchymal Transition. Circ. Res..

[17] Timmerman L.A., Grego-Bessa J., Raya A., Bertrán E., Pérez-Pomares J.M., Díez J., Aranda S., Palomo S., McCormick F., Izpisúa-Belmonte J.C. (2004). Notch Promotes Epithelial-Mesenchymal Transition during Cardiac Development and Oncogenic Transformation. Genes Dev..

[18] Ellis S., Lin E.J., Tartar D. (2018). Immunology of Wound Healing. Curr. Dermatol. Rep..

[19] Hantash B.M., Zhao L., Knowles J.A., Lorenz H.P. (2008). Adult and Fetal Wound Healing. Front. Biosci..

[20] Hu M.S., Maan Z.N., Wu J.C., Rennert R.C., Hong W.X., Lai T.S., Cheung A.T.M., Walmsley G.G., Chung M.T., McArdle A. (2014). Tissue Engineering and Regenerative Repair in Wound Healing. Ann. Biomed. Eng..

[21] Rout-Pitt N., Farrow N., Parsons D., Donnelley M. (2018). Epithelial Mesenchymal Transition (EMT): A Universal Process in Lung Diseases with Implications for Cystic Fibrosis Pathophysiology. Respir. Res..

[22] Qi W., Twigg S., Chen X., Polhill T.S., Poronnik P., Gilbert R.E., Pollock C.A. (2005). Integrated Actions of Transforming Growth Factor-Β1 and Connective Tissue Growth Factor in Renal Fibrosis. Am. J. Physiol. Ren. Physiol..

[23] Li M., Luan F., Zhao Y., Hao H., Zhou Y., Han W., Fu X. (2016). Epithelial-Mesenchymal Transition: An Emerging Target in Tissue Fibrosis. Exp. Biol. Med..

[24] Fintha A., Gasparics Á., Rosivall L., Sebe A. (2019). Therapeutic Targeting of Fibrotic Epithelial-Mesenchymal Transition–an Outstanding Challenge. Front. Pharmacol..

[25] Birchmeier W., Behrens J. (1994). Cadherin Expression in Carcinomas: Role in the Formation of Cell Junctions and the Prevention of Invasiveness. BBA-Rev. Cancer.

[26] Fidler I.J. (1978). Tumor Heterogeneity and the Biology of Cancer Invasion and Metastasis. Cancer Res..

[27] Zolghadr F., Tse N., Loka D., Joun G., Meppat S., Wan V., Zoellner H., Xaymardan M., Farah C.S., Lyons J.G. (2021). A Wnt-Mediated Phenotype Switch along the Epithelial–Mesenchymal Axis Defines Resistance and Invasion Downstream of Ionising Radiation in Oral Squamous Cell Carcinoma. Br. J. Cancer.

[28] Asli N.S., Harvey R.P. (2013). Epithelial to Mesenchymal Transition as a Portal to Stem Cell Characters Embedded in Gene Networks. BioEssays.

[29] Orlichenko L.S., Radisky D.C. (2008). Matrix Metalloproteinases Stimulate Epithelial-Mesenchymal Transition during Tumor Development. Clin. Exp. Metastasis.

[30] Tiwari N., Gheldof A., Tatari M., Christofori G. (2012). EMT as the Ultimate Survival Mechanism of Cancer Cells. Semin. Cancer Biol..

[31] Mitra A., Mishra L., Li S. (2015). EMT, CTCs and CSCs in Tumor Relapse and Drug-Resistance. Oncotarget.

[32] Yan X., Yan L., Liu S., Shan Z., Tian Y., Jin Z. (2015). N-Cadherin, a Novel Prognostic Biomarker, Drives Malignant Progression of Colorectal Cancer. Mol. Med. Rep..

[33] Xue W., Yang L., Chen C., Ashrafizadeh M., Tian Y., Sun R. (2024). Wnt/β-Catenin-Driven EMT Regulation in Human Cancers. Cell. Mol. Life Sci..

[34] Di Croce L., Pelicci P.G. (2003). Tumour-Associated Hypermethylation: Silencing E-Cadherin Expression Enhances Invasion and Metastasis. Eur. J. Cancer.

[35] Mahmood M.Q., Ward C., Muller H.K., Sohal S.S., Walters E.H. (2017). Epithelial Mesenchymal Transition (EMT) and Non-Small Cell Lung Cancer (NSCLC): A Mutual Association with Airway Disease. Med. Oncol..

[36] Krebs A.M., Mitschke J., Losada M.L., Schmalhofer O., Boerries M., Busch H., Boettcher M., Mougiakakos D., Reichardt W., Bronsert P. (2017). The EMT-Activator Zeb1 Is a Key Factor for Cell Plasticity and Promotes Metastasis in Pancreatic Cancer. Nat. Cell Biol..

[37] Blanco M.J., Moreno-Bueno G., Sarrio D., Locascio A., Cano A., Palacios J., Nieto M.A. (2002). Correlation of Snail Expression with Histological Grade and Lymph Node Status in Breast Carcinomas. Oncogene.

[38] Ye X., Tam W.L., Shibue T., Kaygusuz Y., Reinhardt F., Ng Eaton E., Weinberg R.A. (2015). Distinct EMT Programs Control Normal Mammary Stem Cells and Tumour-Initiating Cells. Nature.

[39] Kahlert C., Lahes S., Radhakrishnan P., Dutta S., Mogler C., Herpel E., Brand K., Steinert G., Schneider M., Mollenhauer M. (2011). Overexpression of ZEB2 at the Invasion Front of Colorectal Cancer Is an Independent Prognostic Marker and Regulates Tumor Invasion in Vitro. Clin. Cancer Res..

[40] Lee T.K., Poon R.T.P., Yuen A.P., Ling M.T., Kwok W.K., Wang X.H., Wong Y.C., Guan X., Man K., Chau K.L. (2006). Twist Overexpression Correlates with Hepatocellular Carcinoma Metastasis through Induction of Epithelial-Mesenchymal Transition. Clin. Cancer Res..

[41] Shioiri M., Shida T., Koda K., Oda K., Seike K., Nishimura M., Takano S., Miyazaki M. (2006). Slug Expression Is an Independent Prognostic Parameter for Poor Survival in Colorectal Carcinoma Patients. Br. J. Cancer.

[42] Roth B., Jayaratna I., Sundi D., Cheng T., Melquist J., Choi W., Porten S., Nitti G., Navai N., Wszolek M. (2017). Employing an Orthotopic Model to Study the Role of Epithelialmesenchymal Transition in Bladder Cancer Metastasis. Oncotarget.

[43] Moody S.E., Perez D., Pan T.C., Sarkisian C.J., Portocarrero C.P., Sterner C.J., Notorfrancesco K.L., Cardiff R.D., Chodosh L.A. (2005). The Transcriptional Repressor Snail Promotes Mammary Tumor Recurrence. Cancer Cell.

[44] Gravdal K., Halvorsen O.J., Haukaas S.A., Akslen L.A. (2007). A Switch from E-Cadherin to N-Cadherin Expression Indicates Epithelial to Mesenchymal Transition and Is of Strong and Independent Importance for the Progress of Prostate Cancer. Clin. Cancer Res..

[45] Yang J., Mani S.A., Donaher J.L., Ramaswamy S., Itzykson R.A., Come C., Savagner P., Gitelman I., Richardson A., Weinberg R.A. (2004). Twist, a Master Regulator of Morphogenesis, Plays an Essential Role in Tumor Metastasis. Cell.

[46] Migita T., Ueda A., Ohishi T., Hatano M., Seimiya H., Horiguchi S.I., Koga F., Shibasaki F. (2017). Epithelial-Mesenchymal Transition Promotes SOX2 and NANOG Expression in Bladder Cancer. Lab. Investig..

[47] Ko U.H., Choi J., Choung J., Moon S., Shin J.H. (2019). Physicochemically Tuned Myofibroblasts for Wound Healing Strategy. Sci. Rep..

[48] Barrientos S., Stojadinovic O., Golinko M.S., Brem H., Tomic-Canic M. (2008). Growth Factors and Cytokines in Wound Healing. Wound Repair Regen..

[49] Bongiovanni L., D’Andrea A., Romanucci M., Malatesta D., Candolini M., Salda L.D., Mechelli L., Sforna M., Brachelente C. (2013). Epithelial-to-Mesenchymal Transition: Immunohistochemical Investigation of Related Molecules in Canine Cutaneous Epithelial Tumours. Vet. Dermatol..

[50] Yang M.H., Wu M.Z., Chiou S.H., Chen P.M., Chang S.Y., Liu C.J., Teng S.C., Wu K.J. (2008). Direct Regulation of TWIST by HIF-1α Promotes Metastasis. Nat. Cell Biol..

[51] Simeonov K.P., Byrns C.N., Clark M.L., Norgard R.J., Martin B., Stanger B.Z., Shendure J., McKenna A., Lengner C.J. (2021). Single-Cell Lineage Tracing of Metastatic Cancer Reveals Selection of Hybrid EMT States. Cancer Cell.

[52] Saxena M., Kalathur R.K.R., Neutzner M., Christofori G. (2018). PyMT-1099, a Versatile Murine Cell Model for EMT in Breast Cancer. Sci. Rep..

[53] Pan S., Withnell E., Secrier M. (2024). Classifying Epithelial-Mesenchymal Transition States in Single Cell Cancer Data Using Large Language Models. bioRxiv.

[54] Jolly M.K., Boareto M., Huang B., Jia D., Lu M., Ben-Jacob E., Onuchic J.N., Levine H. (2015). Implications of the hybrid epithelial/mesenchymal phenotype in metastasis. Front. Oncol..

[55] Sinha D., Saha P., Samanta A., Bishayee A. (2020). Emerging concepts of hybrid epithelial-to-mesenchymal transition in cancer metastasis. Biomolecules.

[56] Liao T.T., Yang M.H. (2020). Hybrid epithelial/mesenchymal state in cancer metastasis: Clinical significance and regulatory mechanisms. Cells.

[57] Wu Y., Deng J., Rychahou P.G., Qiu S., Evers B.M., Zhou B.P. (2009). Stabilization of Snail by NF-ΚB Is Required for Inflammation-Induced Cell Migration and Invasion. Cancer Cell.

[58] Bedi U., Mishra V.K., Wasilewski D., Scheel C., Johnsen S.A. (2014). Epigenetic Plasticity: A Central Regulator of Epithelial-Tomesenchymal Transition in Cancer. Oncotarget.

[59] Dongre A., Weinberg R.A. (2019). New Insights into the Mechanisms of Epithelial–Mesenchymal Transition and Implications for Cancer. Nat. Rev. Mol. Cell Biol..

[60] Tan E.J., Olsson A.K., Moustakas A. (2015). Reprogramming during Epithelial to Mesenchymal Transition under the Control of TGFβ. Cell Adh. Migr..

[61] Lin Y.T., Wu K.J. (2020). Epigenetic Regulation of Epithelial-Mesenchymal Transition: Focusing on Hypoxia and TGF-β Signaling. J. Biomed. Sci..

[62] McDonald O.G., Wu H., Timp W., Doi A., Feinberg A.P. (2011). Genome-Scale Epigenetic Reprogramming during Epithelial-to-Mesenchymal Transition. Nat. Struct. Mol. Biol..

[63] Ardizzone A., Bova V., Casili G., Repici A., Lanza M., Giuffrida R., Colarossi C., Mare M., Cuzzocrea S., Esposito E. (2023). Role of Basic Fibroblast Growth Factor in Cancer: Biological Activity, Targeted Therapies, and Prognostic Value. Cells.

[64] Strutz F., Zeisberg M., Ziyadeh F.N., Yang C.Q., Kalluri R., Müller G.A., Neilson E.G., Renziehausen A., Sisic Z. (2002). Role of Basic Fibroblast Growth Factor-2 in Epithelial-Mesenchymal Transformation. Kidney Int..

[65] Lau M.T., So W.K., Leung P.C.K. (2013). Fibroblast Growth Factor 2 Induces E-Cadherin Down-Regulation via PI3K/Akt/MTOR and MAPK/ERK Signaling in Ovarian Cancer Cells. PLoS ONE.

[66] Shirakihara T., Horiguchi K., Miyazawa K., Ehata S., Shibata T., Morita I., Miyazono K., Saitoh M. (2011). TGF-β Regulates Isoform Switching of FGF Receptors and Epithelial-Mesenchymal Transition. EMBO J..

[67] Birchmeier C., Birchmeier W., Gherardi E., Vande Woude G.F. (2003). Met, Metastasis, Motility and More. Nat. Rev. Mol. Cell Biol..

[68] Jiang W.G., Martin T.A., Parr C., Davies G., Matsumoto K., Nakamura T. (2005). Hepatocyte Growth Factor, Its Receptor, and Their Potential Value in Cancer Therapies. Crit. Rev. Oncol. Hematol..

[69] Pothula S.P., Xu Z., Goldstein D., Biankin A.V., Pirola R.C., Wilson J.S., Apte M.V. (2016). Hepatocyte Growth Factor Inhibition: A Novel Therapeutic Approach in Pancreatic Cancer. Br. J. Cancer.

[70] Ogunwobi O.O., Liu C. (2011). Hepatocyte Growth Factor Upregulation Promotes Carcinogenesis and Epithelial-Mesenchymal Transition in Hepatocellular Carcinoma via Akt and COX-2 Pathways. Clin. Exp. Metastasis.

[71] Evans J., Essex A., Xin H., Amitai N., Brinton L., Griner E., Iorns E., Gunn W., Tan F., Lomax J. (2015). Registered Report: Wnt Activity Defines Colon Cancer Stem Cells and Is Regulated by the Microenvironment. eLife.

[72] Yu J., Chen G.G., Lai P.B.S. (2021). Targeting Hepatocyte Growth Factor/c-Mesenchymal–Epithelial Transition Factor Axis in Hepatocellular Carcinoma: Rationale and Therapeutic Strategies. Med. Res. Rev..

[73] Koinis F., Vlachou M.S., Nintos G., Christodoulopoulos G., Panagiotidis E., Eleftheropoulos I., Kallergi G., Samarinas M., Kotsakis A. (2026). The HGF/MET Axis in Advanced Prostate Cancer: From Context-Dependent Biology to Biomarker-Driven Therapeutic Strategies. Cancers.

[74] Davis F.M., Azimi I., Faville R.A., Peters A.A., Jalink K., Putney J.W., Goodhill G.J., Thompson E.W., Roberts-Thomson S.J., Monteith G.R. (2014). Induction of Epithelial-Mesenchymal Transition (EMT) in Breast Cancer Cells Is Calcium Signal Dependent. Oncogene.

[75] Wang Y., Li Y., Wang L., Chen B., Zhu M., Ma C., Mu C., Tao A., Li S., Luo L. (2022). Cinnamaldehyde Suppressed EGF-Induced EMT Process and Inhibits Ovarian Cancer Progression Through PI3K/AKT Pathway. Front. Pharmacol..

[76] Hu Y., Bai J., Zhou D., Zhang L., Chen X., Chen L., Liu Y., Zhang B., Li H., Yin C. (2022). The MiR-4732-5p/XPR1 Axis Suppresses the Invasion, Metastasis, and Epithelial-Mesenchymal Transition of Lung Adenocarcinoma via the PI3K/Akt/GSK3β/Snail Pathway. Mol. Omics.

[77] Zhao L., Li X., Song N., Li A., Hou K., Qu X., Che X., Liu Y. (2018). Src Promotes EGF-Induced Epithelial-to-Mesenchymal Transition and Migration in Gastric Cancer Cells by Upregulating ZEB1 and ZEB2 through AKT. Cell Biol. Int..

[78] Cho K.H., Choi M.J., Jeong K.J., Kim J.J., Hwang M.H., Shin S.C., Park C.G., Lee H.Y. (2014). A ROS/STAT3/HIF-1α Signaling Cascade Mediates EGF-Induced TWIST1 Expression and Prostate Cancer Cell Invasion. Prostate.

[79] Kim J., Kong J., Chang H., Kim H., Kim A. (2016). EGF Induces Epithelial-Mesenchymal Transition through Phospho-Smad2/3-Snail Signaling Pathway in Breast Cancer Cells. Oncotarget.

[80] Ren Y., Hong Y., He W., Liu Y., Chen W., Wen S., Sun M. (2022). EGF/EGFR Promotes Salivary Adenoid Cystic Carcinoma Cell Malignant Neural Invasion via Activation of PI3K/AKT and MEK/ERK Signaling. Curr. Cancer Drug Targets.

[81] Buonato J.M., Lan I.S., Lazzara M.J. (2015). EGF Augments TGFβ-Induced Epithelial-Mesenchymal Transition by Promoting SHP2 Binding to GAB1. J. Cell Sci..

[82] Huang K., Gao N., Bian D., Zhai Q., Yang P., Li M., Wang X. (2020). Correlation between FAK and EGF-Induced EMT in Colorectal Cancer Cells. J. Oncol..

[83] Stewart T.A., Azimi I., Brooks A.J., Thompson E.W., Roberts-Thomson S.J., Monteith G.R. (2016). Janus Kinases and Src Family Kinases in the Regulation of EGF-Induced Vimentin Expression in MDA-MB-468 Breast Cancer Cells. Int. J. Biochem. Cell Biol..

[84] Liu J., Xiao Q., Xiao J., Niu C., Li Y., Zhang X., Zhou Z., Shu G., Yin G. (2022). Wnt/β-Catenin Signalling: Function, Biological Mechanisms, and Therapeutic Opportunities. Signal Transduct. Target. Ther..

[85] Lv J., Cao X.F., Ji L., Zhu B., Wang D.D., Tao L., Li S.Q. (2012). Association of β-Catenin, Wnt1, Smad4, Hoxa9, and Bmi-1 with the Prognosis of Esophageal Squamous Cell Carcinoma. Med. Oncol..

[86] Entezari M., Deldar Abad Paskeh M., Orouei S., Kakavand A., Rezaei S., Sadat Hejazi E., Pashootan P., Nazdari N., Tavakolpournegari A., Hashemi M. (2023). Wnt/β-Catenin Signaling in Lung Cancer: Association with Proliferation, Metastasis, and Therapy Resistance. Curr. Cancer Drug Targets.

[87] Ma F., Li W., Liu C., Li W., Yu H., Lei B., Ren Y., Li Z., Pang D., Qian C. (2017). MiR-23a Promotes TGF-Β1-Induced EMT and Tumor Metastasis in Breast Cancer Cells by Directly Targeting CDH1 and Activating Wnt/β-Catenin Signaling. Oncotarget.

[88] Kwon C., Cheng P., King I.N., Andersen P., Shenje L., Nigam V., Srivastava D. (2011). Notch Post-Translationally Regulates β-Catenin Protein in Stem and Progenitor Cells. Nat. Cell Biol..

[89] Anusewicz D., Orzechowska M., Bednarek A.K. (2021). Notch Signaling Pathway in Cancer—Review with Bioinformatic Analysis. Cancers.

[90] Citarella A., Catanzaro G., Besharat Z.M., Trocchianesi S., Barbagallo F., Gosti G., Leonetti M., Di Fiore A., Coppola L., Autilio T.M. (2023). Hedgehog-GLI and Notch Pathways Sustain Chemoresistance and Invasiveness in Colorectal Cancer and Their Inhibition Restores Chemotherapy Efficacy. Cancers.

[91] Gao Z., Ni X., Zheng B., Sun W., Wan W., Liu H., Ni X., Suo T., Li N., Liu H. (2022). Biliverdin Reductase B Impairs Cholangiocarcinoma Cell Motility by Inhibiting the Notch/Snail Signaling Pathway. J. Cancer.

[92] Hijioka H., Setoguchi T., Miyawaki A., Gao H., Ishida T., Komiya S., Nakamura N. (2010). Upregulation of Notch Pathway Molecules in Oral Squamous Cell Carcinoma. Int. J. Oncol..

[93] Osathanon T., Nowwarote N., Pavasant P. (2016). Expression and Influence of Notch Signalling in Oral Squamous Cell Carcinoma. J. Oral Sci..

[94] Zhang J., Zheng G., Zhou L., Li P.H., Yun M., Shi Q., Wang T., Wu X. (2018). Notch Signalling Induces Epithelial-Mesenchymal Transition to Promote Metastasis in Oral Squamous Cell Carcinoma. Int. J. Mol. Med..

[95] Sahlgren C., Gustafsson M.V., Jin S., Poellinger L., Lendahl U. (2008). Notch Signaling Mediates Hypoxia-Induced Tumor Cell Migration and Invasion. Proc. Natl. Acad. Sci. USA.

[96] Yoo Y.A., Kang M.H., Lee H.J., Kim B.H., Park J.K., Kim H.K., Kim J.S., Oh S.C. (2011). Sonic Hedgehog Pathway Promotes Metastasis and Lymphangiogenesis via Activation of Akt, EMT, and MMP-9 Pathway in Gastric Cancer. Cancer Res..

[97] Riaz S.K., Ke Y., Wang F., Kayani M.A., Malik M.F.A. (2019). Influence of SHH/GLI1 Axis on EMT Mediated Migration and Invasion of Breast Cancer Cells. Sci. Rep..

[98] Bailey J.M., Mohr A.M., Hollingsworth M.A. (2009). Sonic Hedgehog Paracrine Signaling Regulates Metastasis and Lymphangiogenesis in Pancreatic Cancer. Oncogene.

[99] Sui G., Bonde P., Dhara S., Broor A., Wang J., Marti G., Feldmann G., Duncan M., Montgomery E., Maitra A. (2006). Epidermal Growth Factor Receptor and Hedgehog Signaling Pathways Are Active in Esophageal Cancer Cells From Rat Reflux Model. J. Surg. Res..

[100] Zhang J., Tian X.J., Xing J. (2016). Signal Transduction Pathways of EMT Induced by TGF-β, SHH, and WNT and Their Crosstalks. J. Clin. Med..

[101] Li C.W., Xia W., Huo L., Lim S.O., Wu Y., Hsu J.L., Chao C.H., Yamaguchi H., Yang N.K., Ding Q. (2012). Epithelial-Mesenchymal Transition Induced by TNF-α Requires NF-ΚB-Mediated Transcriptional Upregulation of Twist1. Cancer Res..

[102] Zhou X., Wang H., Burg M.B., Ferraris J.D. (2013). Inhibitory Phosphorylation of GSK-3β by AKT, PKA, and PI3K Contributes to High NaCl-Induced Activation of the Transcription Factor NFAT5 (TonEBP/OREBP). Am. J. Physiol. Ren. Physiol..

[103] Ho M.Y., Tang S.J., Chuang M.J., Cha T.L., Li J.Y., Sun G.H., Sun K.H. (2012). TNF-α Induces Epithelial-Mesenchymal Transition of Renal Cell Carcinoma Cells via a GSK3β-Dependent Mechanism. Mol. Cancer Res..

[104] Li H., Zhong A., Li S., Meng X., Wang X., Xu F., Lai M. (2017). The Integrated Pathway of TGFβ/Snail with TNFα/NFκB May Facilitate the Tumor-Stroma Interaction in the EMT Process and Colorectal Cancer Prognosis. Sci. Rep..

[105] Semenza G.L. (2016). The Hypoxic Tumor Microenvironment: A Driving Force for Breast Cancer Progression. Biochim. Biophys. Acta Mol. Cell Res..

[106] Zhang X., Sai B., Wang F., Wang L., Wang Y., Zheng L., Li G., Tang J., Xiang J. (2019). Hypoxic BMSC-Derived Exosomal MiRNAs Promote Metastasis of Lung Cancer Cells via STAT3-Induced EMT. Mol. Cancer.

[107] Zou C., Zhu J., Xiong J., Tian Y., Peng Y., Cheung E., Zhang D. (2024). Comprehensive Characterization of the Integrin Family Across 32 Cancer Types. Genom. Proteom. Bioinform..

[108] Su C.Y., Li J.Q., Zhang L.L., Wang H., Wang F.H., Tao Y.W., Wang Y.Q., Guo Q.R., Li J.J., Liu Y. (2020). The Biological Functions and Clinical Applications of Integrins in Cancers. Front. Pharmacol..

[109] Kariya Y., Nishita M. (2025). Integrins in Cancer Drug Resistance: Molecular Mechanisms and Clinical Implications. Int. J. Mol. Sci..

[110] Bandyopadhyay A., Raghavan S. (2009). Defining the Role of Integrin Alphavbeta6 in Cancer. Curr. Drug Targets.

[111] Mori S., Kodaira M., Ito A., Okazaki M., Kawaguchi N., Hamada Y., Takada Y., Matsuura N. (2015). Enhanced Expression of Integrin Aνβ3 Induced by TGF-β Is Required for the Enhancing Effect of Fibroblast Growth Factor 1 (FGF1) in TGF-β-Induced Epithelial-Mesenchymal Transition (EMT) in Mammary Epithelial Cells. PLoS ONE.

[112] Bogdanović B., Fagret D., Ghezzi C., Montemagno C. (2024). Integrin Targeting and Beyond: Enhancing Cancer Treatment with Dual-Targeting RGD (Arginine–Glycine–Aspartate) Strategies. Pharmaceuticals.

[113] Hou J., Yan D., Liu Y., Huang P., Cui H. (2020). The Roles of Integrin A5Β1 in Human Cancer. Onco Targets. Ther..

[114] Gu Y., Dong B., He X., Qiu Z., Zhang J., Zhang M., Liu H., Pang X., Cui Y. (2023). The Challenges and Opportunities of Avβ3-Based Therapeutics in Cancer: From Bench to Clinical Trials. Pharmacol. Res..

[115] Echavidre W., Picco V., Faraggi M., Montemagno C. (2022). Integrin-Avβ3 as a Therapeutic Target in Glioblastoma: Back to the Future?. Pharmaceutics.

[116] Franovic A., Elliott K.C., Seguin L., Camargo M.F., Weis S.M., Cheresh D.A. (2015). Glioblastomas Require Integrin Avβ3/PAK4 Signaling to Escape Senescence. Cancer Res..

[117] Poyyakkara A., Raji G.R., Padmaja K.P., Ramachandran V., Changmai U., Edatt L., Punathil R., Kumar V.B.S. (2023). Integrin Β4 Induced Epithelial-to-Mesenchymal Transition Involves MiR-383 Mediated Regulation of GATA6 Levels. Mol. Biol. Rep..

[118] Nam E.H., Lee Y., Park Y.K., Lee J.W., Kim S. (2012). Zeb2 Upregulates Integrin A5 Expression through Cooperation with Sp1 to Induce Invasion during Epithelial-Mesenchymal Transition of Human Cancer Cells. Carcinogenesis.

[119] Harryman W.L., Marr K.D., Nagle R.B., Cress A.E. (2022). Integrins and Epithelial-Mesenchymal Cooperation in the Tumor Microenvironment of Muscle-Invasive Lethal Cancers. Front. Cell Dev. Biol..

[120] Li S., Sampson C., Liu C., Piao H., Liu H.X. (2023). Integrin Signaling in Cancer: Bidirectional Mechanisms and Therapeutic Opportunities. Cell Commun. Signal..

[121] Mamuya F.A., Duncan M.K. (2012). AV Integrins and TGF-β-Induced EMT: A Circle of Regulation. J. Cell. Mol. Med..

[122] Matsuoka T., Yashiro M. (2023). The Role of the Transforming Growth Factor-β Signaling Pathway in Gastrointestinal Cancers. Biomolecules.

[123] Subbaram S., Dipersio C.M. (2011). Integrin A3β1 as a Breast Cancer Target. Expert Opin. Ther. Targets.

[124] Oloumi A., McPhee T., Dedhar S. (2004). Regulation of E-Cadherin Expression and β-Catenin/Tcf Transcriptional Activity by the Integrin-Linked Kinase. Biochim. Biophys. Acta Mol. Cell Res..

[125] Khalili-Tanha G., Radisky E.S., Radisky D.C., Shoari A. (2025). Matrix Metalloproteinase-Driven Epithelial-Mesenchymal Transition: Implications in Health and Disease. J. Transl. Med..

[126] Wu W.S., You R.I., Cheng C.C., Lee M.C., Lin T.Y., Hu C.T. (2017). Snail Collaborates with EGR-1 and SP-1 to Directly Activate Transcription of MMP 9 and ZEB1. Sci. Rep..

[127] Miyoshi A., Kitajima Y., Kido S., Shimonishi T., Matsuyama S., Kitahara K., Miyazaki K. (2005). Snail Accelerates Cancer Invasion by Upregulating MMP Expression and Is Associated with Poor Prognosis of Hepatocellular Carcinoma. Br. J. Cancer.

[128] Radisky D.C., Levy D.D., Littlepage L.E., Liu H., Nelson C.M., Fata J.E., Leake D., Godden E.L., Albertson D.G., Nieto M.A. (2005). Rac1b and Reactive Oxygen Species Mediate MMP-3-Induced EMT and Genomic Instability. Nature.

[129] Lin C.Y., Tsai P.H., Kandaswami C.C., Lee P.P., Huang C.J., Hwang J.J., Lee M.T. (2011). Matrix Metalloproteinase-9 Cooperates with Transcription Factor Snail to Induce Epithelial-Mesenchymal Transition. Cancer Sci..

[130] Ardalan Khales S., Abbaszadegan M.R., Majd A., Forghanifard M.M. (2020). TWIST1 Upregulates Matrix Metalloproteinase (MMP) Genes Family in Esophageal Squamous Carcinoma Cells. Gene Expr. Patterns.

[131] Bae G.Y., Choi S.J., Lee J.S., Jo J., Lee J., Kim J., Cha H.J. (2013). Loss of E-Cadherin Activates EGFR-MEK/ERK Signaling, Which Promotes Invasion via the ZEB1/MMP2 Axis in Non-Small Cell Lung Cancer. Oncotarget.

[132] Wan R., Mo Y., Chien S., Li Y., Li Y., Tollerud D.J., Zhang Q. (2011). The Role of Hypoxia Inducible Factor-1α in the Increased MMP-2 and MMP-9 Production by Human Monocytes Exposed to Nickel Nanoparticles. Nanotoxicology.

[133] Ito K., Kitajima Y., Kai K., Matsufuji S., Yamada K., Egawa N., Kitagawa H., Okuyama K., Tanaka T., Noshiro H. (2021). Matrix Metalloproteinase-1 Expression Is Regulated by HIF-1-dependent and Epigenetic Mechanisms and Serves a Tumor-suppressive Role in Gastric Cancer Progression. Int. J. Oncol..

[134] Lan Y., Zhao S., Hou T., Ren Y., Tang J., Yin S., Wu Y. (2024). Mechanism of HIF-1α Promoting Proliferation, Invasion and Metastasis of Nasopharyngeal Carcinoma by Regulating MMP-2 in Hypoxic Microenvironment. Heliyon.

[135] Xia P. (2026). The Significance of Epithelial–Mesenchymal Transition (EMT) in the Initiation, Plasticity, and Treatment of Glioblastoma. Genes Dis..

[136] Chang J.H., Huang Y.H., Cunningham C.M., Han K.Y., Chang M., Seiki M., Zhou Z., Azar D.T. (2016). Matrix Metalloproteinase 14 Modulates Signal Transduction and Angiogenesis in the Cornea. Surv. Ophthalmol..

[137] Wang Y., Wei Y., Huang J., Li X., You D., Wang L., Ma X. (2024). Prognostic Value of Matrix Metalloproteinase-2 Protein and Matrix Metalloproteinase-9 Protein in Colorectal Cancer: A Meta-Analysis. BMC Cancer.

[138] Scheau C., Badarau I.A., Costache R., Caruntu C., Mihai G.L., Didilescu A.C., Constantin C., Neagu M. (2019). The Role of Matrix Metalloproteinases in the Epithelial-Mesenchymal Transition of Hepatocellular Carcinoma. Anal. Cell. Pathol..

[139] Rigoulet M., Bouchez C.L., Paumard P., Ransac S., Cuvellier S., Duvezin-Caubet S., Mazat J.P., Devin A. (2020). Cell Energy Metabolism: An Update. Biochim. Biophys. Acta Bioenerg..

[140] Schiliro C., Firestein B.L. (2021). Mechanisms of Metabolic Reprogramming in Cancer Cells Supporting Enhanced Growth and Proliferation. Cells.

[141] Lee S., Tak E., Lee J., Rashid M., Murphy M.P., Ha J., Kim S.S. (2011). Mitochondrial H2 O2 Generated from Electron Transport Chain Complex i Stimulates Muscle Differentiation. Cell Res..

[142] Barba I., Carrillo-Bosch L., Seoane J. (2024). Targeting the Warburg Effect in Cancer: Where Do We Stand?. Int. J. Mol. Sci..

[143] Fadaka A., Ajiboye B., Ojo O., Adewale O., Olayide I., Emuowhochere R. (2017). Biology of Glucose Metabolization in Cancer Cells. J. Oncol. Sci..

[144] Sieber-Frank J., Stark H.J., Kalteis S., Prigge E.S., Köhler R., Andresen C., Henkel T., Casari G., Schubert T., Fischl W. (2021). Treatment Resistance Analysis Reveals GLUT-1-Mediated Glucose Uptake as a Major Target of Synthetic Rocaglates in Cancer Cells. Cancer Med..

[145] Chen C.L., Lin C.Y., Kung H.J. (2021). Targeting Mitochondrial Oxphos and Their Regulatory Signals in Prostate Cancers. Int. J. Mol. Sci..

[146] Yao C.H., Wang R., Wang Y., Kung C.P., Weber J.D., Patti G.J. (2019). Mitochondrial Fusion Supports Increased Oxidative Phosphorylation during Cell Proliferation. eLife.

[147] Passaniti A., Kim M.S., Polster B.M., Shapiro P. (2022). Targeting Mitochondrial Metabolism for Metastatic Cancer Therapy. Mol. Carcinog..

[148] Cannata D., Fierz Y., Vijayakumar A., LeRoith D. (2010). Type 2 Diabetes and Cancer: What Is the Connection?. Mt. Sinai J. Med..

[149] Shahid R.K., Ahmed S., Le D., Yadav S. (2021). Diabetes and Cancer: Risk, Challenges, Management and Outcomes. Cancers.

[150] Lien K.H., Padua P.F.C., Tay Z.Y., Kao H.K., Hung S.Y., Huang Y., Tsang N.M., Chang K.P. (2020). Influence of Hyperglycemia on Treatment Outcomes of Oral Cavity Squamous Cell Carcinoma. J. Oral Maxillofac. Surg..

[151] Ramos-Garcia P., Roca-Rodriguez M.d.M., Aguilar-Diosdado M., Gonzalez-Moles M.A. (2021). Diabetes Mellitus and Oral Cancer/Oral Potentially Malignant Disorders: A Systematic Review and Meta-Analysis. Oral Dis..

[152] Tay Z.Y., Kao H.K., Lien K.H., Hung S.Y., Huang Y., Tsang N.M., Chang K.P. (2020). The Impact of Preoperative Glycated Hemoglobin Levels on Outcomes in Oral Squamous Cell Carcinoma. Oral Dis..

[153] De Paz D., Kao H.K., Huang Y., Chang K.P. (2017). Prognostic Stratification of Patients With Advanced Oral Cavity Squamous Cell Carcinoma. Curr. Oncol. Rep..

[154] Li W., Liu H., Qian W., Cheng L., Yan B., Han L., Xu Q., Ma Q., Ma J. (2018). Hyperglycemia Aggravates Microenvironment Hypoxia and Promotes the Metastatic Ability of Pancreatic Cancer. Comput. Struct. Biotechnol. J..

[155] Pizzino G., Irrera N., Cucinotta M., Pallio G., Mannino F., Arcoraci V., Squadrito F., Altavilla D., Bitto A. (2017). Oxidative Stress: Harms and Benefits for Human Health. Oxid. Med. Cell. Longev..

[156] Reczek C.R., Chandel N.S. (2017). The Two Faces of Reactive Oxygen Species in Cancer. Annu. Rev. Cancer Biol..

[157] Mossenta M., Busato D., Dal Bo M., Toffoli G. (2020). Glucose Metabolism and Oxidative Stress in Hepatocellular Carcinoma: Role and Possible Implications in Novel Therapeutic Strategies. Cancers.

[158] Reinfeld B.I., Rathmell W.K., Kim T.K., Rathmell J.C. (2022). The Therapeutic Implications of Immunosuppressive Tumor Aerobic Glycolysis. Cell. Mol. Immunol..

[159] Loponte S., Lovisa S., Deem A.K., Carugo A., Viale A. (2019). The Many Facets of Tumor Heterogeneity: Is Metabolism Lagging Behind?. Cancers.

[160] Kang H., Kim H., Lee S., Youn H., Youn B. (2019). Role of Metabolic Reprogramming in Epithelial–Mesenchymal Transition (EMT). Int. J. Mol. Sci..

[161] Liberti M.V., Locasale J.W. (2016). The Warburg Effect: How Does It Benefit Cancer Cells?. Trends Biochem. Sci..

[162] Duan W., Shen X., Lei J., Xu Q., Yu Y., Li R., Wu E., Ma Q. (2014). Hyperglycemia, a Neglected Factor during Cancer Progression. BioMed Res. Int..

[163] Jo H., Lee J., Jeon J., Kim S.Y., Chung J.I., Ko H.Y., Lee M., Yun M. (2020). The Critical Role of Glucose Deprivation in Epithelial-Mesenchymal Transition in Hepatocellular Carcinoma under Hypoxia. Sci. Rep..

[164] Hahn J.-S., Hu Z., Thiele D.J., Iyer V.R. (2004). Genome-Wide Analysis of the Biology of Stress Responses through Heat Shock Transcription Factor. Mol. Cell. Biol..

[165] Kim N.H., Cha Y.H., Lee J., Lee S.H., Yang J.H., Yun J.S., Cho E.S., Zhang X., Nam M., Kim N. (2017). Snail Reprograms Glucose Metabolism by Repressing Phosphofructokinase PFKP Allowing Cancer Cell Survival under Metabolic Stress. Nat. Commun..

[166] Wang Z., Li Y., Sarkar F. (2010). Signaling Mechanism(S) of Reactive Oxygen Species in Epithelial-Mesenchymal Transition Reminiscent of Cancer Stem Cells in Tumor Progression. Curr. Stem Cell Res. Ther..

[167] Li W., Zhang L., Chen X., Jiang Z., Zong L., Ma Q. (2016). Hyperglycemia Promotes the Epithelial-Mesenchymal Transition of Pancreatic Cancer via Hydrogen Peroxide. Oxid. Med. Cell. Longev..

[168] Liu D., Sun L., Qin X., Liu T., Zhang S., Liu Y., Li S., Guo K. (2016). HSF1 Promotes the Inhibition of EMT-Associated Migration by Low Glucose via Directly Regulating Snail1 Expression in HCC Cells. Discov. Med..

[169] Xu X., Chen B., Zhu S., Zhang J., He X., Cao G., Chen B. (2019). Hyperglycemia Promotes Snail-Induced Epithelial-Mesenchymal Transition of Gastric Cancer via Activating ENO1 Expression. Cancer Cell Int..

[170] Zielinska H.A., Holly J.M.P., Bahl A., Perks C.M. (2018). Inhibition of FASN and ERα Signalling during Hyperglycaemia-Induced Matrix-Specific EMT Promotes Breast Cancer Cell Invasion via a Caveolin-1-Dependent Mechanism. Cancer Lett..

[171] Li S., Zhu H., Chen H., Xia J., Zhang F., Xu R., Lin Q. (2020). Glucose Promotes Epithelial-Mesenchymal Transitions in Bladder Cancer by Regulating the Functions of YAP1 and TAZ. J. Cell. Mol. Med..

[172] Gu C.J., Xie F., Zhang B., Yang H.L., Cheng J., He Y.Y., Zhu X.Y., Li D.J., Li M.Q. (2018). High Glucose Promotes Epithelial-Mesenchymal Transition of Uterus Endometrial Cancer Cells by Increasing ER/GLUT4-Mediated VEGF Secretion. Cell. Physiol. Biochem..

[173] Hua W., ten Dijke P., Kostidis S., Giera M., Hornsveld M. (2020). TGFβ-Induced Metabolic Reprogramming during Epithelial-to-Mesenchymal Transition in Cancer. Cell. Mol. Life Sci..

[174] Acquaviva J., Wong R., Charest A. (2009). The Multifaceted Roles of the Receptor Tyrosine Kinase ROS in Development and Cancer. Biochim. Biophys. Acta Rev. Cancer.

[175] Liu F., Matsuura I. (2005). Inhibition of Smad Antiproliferative Function by CDK Phosphorylation. Cell Cycle.

[176] Lu J., Li P., Yu D. (2010). Abstract 5760: 14-3-3zeta Cooperates with ErbB2 to Promote Ductal Carcinoma in Situ Progression to Invasive Breast Cancer by Inducing Epithelial-Mesenchymal Transition. Cancer Res..

[177] Yeh H.W., Hsu E.C., Lee S.S., Lang Y.D., Lin Y.C., Chang C.Y., Lee S.Y., Gu D.L., Shih J.H., Ho C.M. (2018). PSPC1 Mediates TGF-Β1 Autocrine Signalling and Smad2/3 Target Switching to Promote EMT, Stemness and Metastasis. Nat. Cell Biol..

[178] Inoki K., Haneda M., Maeda S., Koya D., Kikkawa R. (1999). TGF-Β1 Stimulates Glucose Uptake by Enhancing GLUT1 Expression in Mesangial Cells. Kidney Int..

[179] Li W., Wei Z., Liu Y., Li H., Ren R., Tang Y. (2010). Increased 18F-FDG Uptake and Expression of Glut1 in the EMT Transformed Breast Cancer Cells Induced by TGF-β. Neoplasma.

[180] Masin M., Vazquez J., Rossi S., Groeneveld S., Samson N., Schwalie P.C., Deplancke B., Frawley L.E., Gouttenoire J., Moradpour D. (2014). GLUT3 Is Induced during Epithelial-Mesenchymal Transition and Promotes Tumor Cell Proliferation in Non-Small Cell Lung Cancer. Cancer Metab..

[181] Nilchian A., Giotopoulou N., Sun W., Fuxe J. (2020). Different Regulation of Glut1 Expression and Glucose Uptake during the Induction and Chronic Stages of TGFβ1-Induced EMT in Breast Cancer Cells. Biomolecules.

[182] Pehlivanoglu S., Sahan O.B., Pehlivanoglu S., Aktas Kont K. (2021). Epithelial Mesenchymal Transition Regulator TWIST1 Transcription Factor Stimulates Glucose Uptake through Upregulation of GLUT1, GLUT3, and GLUT12 in Vitro. In Vitro Cell. Dev. Biol. Anim..

[183] Colvin H., Nishida N., Konno M., Haraguchi N., Takahashi H., Nishimura J., Hata T., Kawamoto K., Asai A., Tsunekuni K. (2016). Oncometabolite D-2-Hydroxyglurate Directly Induces Epithelial-Mesenchymal Transition and Is Associated with Distant Metastasis in Colorectal Cancer. Sci. Rep..

[184] Kolosionek E., Savai R., Ghofrani H.A., Weissmann N., Guenther A., Grimminger F., Seeger W., Banat G.A., Schermuly R.T., Pullamsetti S.S. (2009). Expression and Activity of Phosphodiesterase Isoforms during Epithelial Mesenchymal Transition: The Role of Phosphodiesterase 4. Mol. Biol. Cell.

[185] Ramesh V., Brabletz T., Ceppi P. (2020). Targeting EMT in Cancer with Repurposed Metabolic Inhibitors. Trends Cancer.

[186] Schacke M., Kumar J., Colwell N., Hermanson K., Folle G.A., Nechaev S., Dhasarathy A., Lafon-Hughes L. (2019). PARP-1/2 Inhibitor Olaparib Prevents or Partially Reverts EMT Induced by TGF-β in NMuMG Cells. Int. J. Mol. Sci..

[187] Wu Q.-G., Zhang M.-J., Lan Y.-B., Ma C.-L., Fu W.-J. (2025). Hyperglycemia-Induced Overexpression of CREB3L3 Promotes the Epithelial-to-Mesenchymal Transition in Bladder Urothelial Cells in Diabetes Mellitus. World J. Diabetes.

[188] Liu Z., Hayashi H., Matsumura K., Ogata Y., Sato H., Shiraishi Y., Uemura N., Miyata T., Higashi T., Nakagawa S. (2023). Hyperglycaemia induces metabolic reprogramming into a glycolytic phenotype and promotes epithelial-mesenchymal transitions via YAP/TAZ-Hedgehog signalling axis in pancreatic cancer. Br. J. Cancer.

[189] Lu Q., Wang W.W., Zhang M.Z., Ma Z.X., Qiu X.R., Shen M., Yin W.W. (2019). ROS induces epithelial-mesenchymal transition via the TGF-β1/PI3K/Akt/mTOR pathway in diabetic nephropathy. Exp. Ther. Med..

[190] Viedma-Rodríguez A.R., Martínez-Hernández M., Flores-López L., Velázquez-Flores M., Esparza-Garrido R., Prado-Baeza J., Baiza-Gutman L. (2025). High glucose promotes cisplatin chemoresistance in MDA-MB-231 breast cancer derived cells through changes in gene expression and multiple signaling pathways. Biomed. Rep..

